# Neuroprotective Mechanism of Icariin on Hypoxic Ischemic Brain Damage in Neonatal Mice

**DOI:** 10.1155/2022/1330928

**Published:** 2022-11-15

**Authors:** Mengxia Wang, Xiaoxia Yang, Qian Zhou, Yingqi Guo, Yingxiu Chen, Linyang Song, Junhua Yang, Lixia Li, Li Luo

**Affiliations:** ^1^Intensive Care Unit, Guangdong Second Provincial General Hospital, Guangzhou 510317, China; ^2^School of Biosciences & Biopharmaceutics, Guangdong Pharmaceutical University, Guangzhou 510006, China; ^3^Guangdong Medical Association, Guangzhou 510180, China

## Abstract

**Objective:**

Our previous results showed that icariin (ICA) could inhibit apoptosis and provide neuroprotection against hypoxic-ischemic brain damage (HIBD) in neonatal mice, but the specific mechanism of its neuroprotective effect remains unknown. This study aims at exploring whether ICA plays a neuroprotective role in apoptosis inhibition by regulating autophagy through the estrogen receptor *α* (ER*α*)/estrogen receptor *β* (ER*β*) pathway in neonatal mice with HIBD.

**Methods:**

A neonatal mouse model of HIBD was constructed in vivo, and an oxygen and glucose deprivation (OGD) model in HT22 cells from the hippocampal neuronal system was constructed in vitro. The effects of ICA pretreatment on autophagy and the expression of ER*α* and ER*β* were detected in vitro and in vivo, respectively. ICA pretreatment was also supplemented with the autophagy inhibitor 3-methyladenine (3-MA), ER*α* inhibitor methylpiperidino pyrazole (MPP), and ER*β* inhibitor 4-(2-phenyl-5,7-bis (trifluoromethyl) pyrazolo [1,5-a] pyramidin-3-yl) phenol (PHTPP) to further detect whether ICA pretreatment can activate the ER*α*/ER*β* pathway to promote autophagy and reduce HIBD-induced apoptosis to play a neuroprotective role against HIBD in neonatal mice.

**Results:**

ICA pretreatment significantly promoted autophagy in HIBD mice. Treatment with 3-MA significantly inhibited the increase in autophagy induced by ICA pretreatment, reversed the neuroprotective effect of ICA pretreatment, and promoted apoptosis. Moreover, ICA pretreatment significantly increased the expression levels of the ER*α* and ER*β* proteins in HIBD newborn mice. Both MPP and PHTPP administration significantly inhibited the expression levels of the ER*α* and ER*β* proteins activated by ICA pretreatment, reversed the neuroprotective effects of ICA pretreatment, inhibited the increase in autophagy induced by ICA pretreatment, and promoted apoptosis.

**Conclusion:**

ICA pretreatment may promote autophagy by activating the ER*α* and ER*β* pathways, thus reducing the apoptosis induced by HIBD and exerting a neuroprotective effect on neonatal mice with HIBD.

## 1. Introduction

Hypoxic-ischemic brain damage (HIBD) is caused by decreases in both oxygen flow to the brain and cerebral blood flow [1], and it is one of the main causes of acute death and neurodevelopmental disorders in neonates [2, 3]. In developed countries, the incidence of HIBD is 1 to 8 per 1000 neonates, while in developing countries, the incidence of HIBD is as high as 26 per 1000 neonates [4–7]. Neonatal HIBD accounts for 23% of global neonatal deaths, and survivors may suffer from permanent neurological conditions, including cerebral palsy, epilepsy, mental retardation, and hearing and vision disorders [2, 8–10]. Therapeutic hypothermia (TH) is the only established treatment option that reduces the risk of infant death and neurodevelopmental disorders in HIBD [11]. Unfortunately, the treatment window of neonatal HIBD is only 6 h, and TH treatment cannot be started in such a short time. Even if TH treatment is adopted, its benefits will gradually diminish with the passage of time, thus leading to the death of nearly half of infants with moderate to severe HIBD or their development of severe neurodevelopmental disorders [12]. In addition, TH treatment has not been successful in reducing neonatal mortality owing to the high cost of treatment, which limits its introduction in low- and middle-income countries with high rates of HIBD. Therefore, it is urgent to deeply explore the pathogenesis of neonatal HIBD and find new therapeutic interventions that can help to improve brain injury and lifetime neurological sequelae.

**Figure 1:**
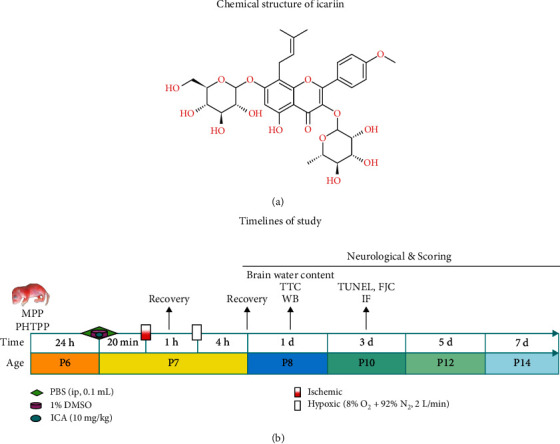
Molecular structure and timelines of the study. (a) Molecular structure of ICA. (b) Timeline for the in vivo studies. *P* represents the number of days after mouse birth.

Studies have shown that icariin (ICA, molecular formula: C_33_H_40_O_5_, molecular weight: 676.66, purity > 98%, Figure 1(a)) is a flavonoid glycoside extracted from the medicinal plant *Epimedium* of Berberidaceae and the main active ingredient of *Epimedium*. It can enter the brain tissue of animals through the blood–brain barrier to play a neuroprotective role and has multiple pharmacological effects, such as antiapoptotic, anti-inflammatory, antioxidative, immune enhancing, and endocrine regulating properties [13–16]. Our research group has indicated in previous studies that in neonatal mice with HIBD, ICA pretreatment inhibits apoptosis and exerts a neuroprotective effect, but the specific mechanism of its neuroprotective effect in HIBD remains unknown [17].

At present, studies have revealed that the pathogenesis of HIBD mainly includes autophagy, apoptosis, endoplasmic reticulum stress, and neuroexcitatory toxicity [18]. Among them, autophagy and apoptosis are molecular mechanisms that are closely related in a variety of cell stresses. Apoptosis is an autonomic and orderly process of cell death that plays an important role in various central nervous system diseases. Apoptosis leads to the delayed death of a large number of brain cells, neuronal loss, and neurodegeneration, playing an important role in maintaining the dynamic balance in the cell and the body's growth and development [19, 20]. For example, in a cerebral ischemia/reperfusion (I/R) model, kaempferol exerted neuroprotective effects on cerebral I/R injury by inhibiting oxidative stress and inflammation-induced apoptosis [21]. Colony-stimulating factor 1 can inhibit neuronal apoptosis and reduce the nerve damage caused by neonatal hypoxic-ischemic encephalopathy (HIE) by regulating the colony-stimulating factor 1 receptor (CSF1R)/phospholipase C gamma 2 (PLCG2)/protein kinase A system (PKA)/uncoupling protein 2 (UCP2) signaling pathway [22]. In addition, curcumin can promote autophagy of nucleus pulposus cells through the AMP-activated protein kinase (AMPK)/mammalian target of rapamycin (mTOR)/autophagy-related protein 1 homolog (ULK1) signaling pathway and thereby inhibit nucleus pulposus cell apoptosis to prevent disc degeneration [23]. Apoptosis plays an important role in the pathophysiological process of HIBD. In the process of neonatal HIBD and the decrease in cerebral blood flow, an excess of free radicals, calcium overload, and excitatory toxicity will be produced, which will induce neuronal apoptosis and aggravate the occurrence and development of HIBD [24]. Therefore, targeting key apoptosis regulators to block hypoxia-ischemia (HI)-induced apoptosis has become a key process in the neurological outcome after HIBD. This finding was consistent with the previous finding of our research group that ICA pretreatment inhibited apoptosis and played a neuroprotective role in neonatal mouse HIBD [17].

Apoptosis and autophagy are two closely related processes. Autophagy is an evolutionarily conserved lysosomal degradation process of intracellular substance turnover, aiming to degrade spacers, misfolded proteins, damaged or aged organelles, and mutant proteins to maintain intracellular circulation and homeostasis [25, 26]. It has been reported that autophagy is involved in the occurrence and development of various neurodegenerative diseases. In Alzheimer's disease (AD), the CCZ1-MON1A-RAB7 complex can reduce neurological dysfunction and memory impairment in AD mice by enhancing autophagy [27]. In Parkinson's disease, the activation of autophagy helps to improve *α*-synuclein-induced tyrosine hydroxylase loss and behavioral deficits [28]. In recent years, studies have shown that autophagy is closely related to many ischemic diseases. In a cerebral I/R model, a tissue-type plasminogen activator protects mouse neurons from I/R injury by regulating mitochondrial autophagy [29]. In acute renal I/R injury, enhanced renal tubule autophagy and inhibition of renal tubule apoptosis in SD rats can improve acute renal I/R injury [30]. However, excessive and long-term autophagy may lead to cell death, known as autophagic cell death [31–33]. Therefore, the occurrence of autophagy is usually accompanied by apoptosis, which is interdependent, antagonistic, and interconverted.

Previous studies have shown that the activation of autophagy helps to improve the pathological damage caused by neurodegenerative and ischemic diseases. Autophagy induction has been found in both neonatal and adult rodents after HIBD, but its role in HIE is still controversial [7, 34]. It has been reported that inhibition of autophagy aggravates neuronal mitochondrial dysfunction and aggravates apoptosis in HIBD pups [35]. Rapamycin treatment significantly blocked the nicotine-mediated downregulation of autophagy and alleviated cerebral infarction injury in HIBD newborn rats, thereby playing a neuroprotective role [36]. However, according to the research by Melk et al., autophagy may be a detrimental factor in the development of HIBD in neonates; additionally, glycine exerts a neuroprotective effect on HIBD by reducing mitochondrial-mediated autophagy [37]. The difference may be determined by the animal species, the degree of autophagy induction, and the duration of autophagy activation [31]. Therefore, determining the possible mechanism of apoptosis and autophagy changes after HIBD and targeting autophagy regulators to inhibit apoptosis may be an effective strategy for the treatment of neonatal HIBD.

To further analyze the regulatory mechanism of autophagy in HIBD, we examined the estrogen receptor (ER) in the literature. Estrogen is a cholesterol-derived hormone formed by the conversion of testosterone into an androgen that is catalyzed by estrogen synthase, which can be used as a powerful neuroprotective factor against inflammation, oxidation, apoptosis, and vasodilation in animals with brain injury [38, 39]. Chrysin has been shown to play a neuroprotective role against cerebral I/R injury in male rats by modulating estrogen [40]. Resveratrol reduced neurotoxin-induced cognitive impairment by regulating the hippocampal estrogen-N-methyl-D-aspartate receptor- (NMDAR-) brain-derived neurotrophic factor (BDNF) signaling pathway in female mice [41]. Natural estrogens mainly include estrone (E1), estradiol (E2), and estriol (E3). Among them, E2 is the most abundant estrogen with the strongest activity, which has significant neuroprotective effects on different kinds of brain injury [42]. Studies have shown that E2 plays a neuroprotective role in ischemic neuron injury by inhibiting the expression and function of acid-sensitive ion channel proteins in an ischemic stroke model [43]. In addition, intranasal administration of E2-loaded gelatin nanoparticles effectively improved cerebral ischemic injury in mice and provided neuroprotection [44]. E2 mainly activates the ER to exert neuroprotective and cognitive repair effects on brain injury by various mechanisms. The ER has two forms, estrogen receptor *α* (ER*α*) and estrogen receptor *β* (ER*β*), and is widely distributed in the hippocampus, cerebral cortex and basal forebrain, and other brain regions related to learning and memory [45, 46]. In experiments HIBD with animals, the expression levels of ER*α* and ER*β* are dramatically decreased, and the ER inhibitor ICI-182780 reverses the neuroprotective effect of the neuroprotective drug notoginsenoside R1 on neonatal HIBD and aggravates neuronal apoptosis [47]. Additionally, in microglia cultured in vitro, ERs play a neuroprotective role by inhibiting Toll-like receptor 4- (TLR4-) mediated microglial inflammation [48]. In atherosclerotic diseases, estrogen can reduce inflammation and endothelial cell apoptosis and prevent atherosclerosis by upregulating ER-activated autophagy, indicating that there is a positive regulatory relationship between the ER and autophagy [49]. However, whether ER*α* and ER*β* can improve neonatal HIBD by regulating autophagy to inhibit apoptosis in a neonatal HIBD model is unknown.

A previous study by our group found that ICA pretreatment inhibited apoptosis and played a neuroprotective role in HIBD in newborn mice [17]. The mechanism of ICA as a neuroprotective drug has been partially reported; for example, ICA can promote autophagy in the cerebral cortex and hippocampus to improve brain function decline in aging rats through the AMPK/mTOR/ULK1 signaling pathway [50]. ICA, similar to an 8-prenyl flavonol glycoside compound extracted from Herba *Epimedii*, has strong estrogen-like activity and can promote estrogen biosynthesis [51, 52]. ICA inhibits osteoblast apoptosis by activating ER*α* signaling [53] and exerts estrogen-like activity through ER*β* to improve mouse encephalomyelitis [54]. Therefore, we hypothesized that ICA may promote autophagy by activating the ER*α* and ER*β* pathways and thus reduce HIBD-induced apoptosis to play a neuroprotective role in neonatal mice with HIBD.

## 2. Materials and Methods

In this experiment, our scientific assumptions were firstly verified by in vivo experiments. After the confirmed results were obtained from in vivo experiments, we further verified by in vitro experiments. The following materials and methods are descriptions of in vivo experiments. Please refer to the Supplementary material—Materials and Methods of in vitro experiments.

### 2.1. Experimental Animals

Animal procedures were approved by the Laboratory Animal Committee of Guangdong Pharmaceutical University (license number: gdpulac2019005), and all animal experimental protocols conformed to the National Institutes of Health (NIH) guidelines for the care and use of laboratory animals. A total of 306 7-day-old (P7) C57BL/6 neonatal mice (3-5 g, regardless of sex) were used in this study. All experimental animals were provided by Guangdong Medical Laboratory Animal Center (Permit No. SYXK [Yue] 2018−0002). All animals were reared on a 12 h day/12 h night cycle at a temperature of 25 ± 2°C with a relative humidity of 60%-80% under specific pathogen-free conditions. To avoid adverse effects of diet on the results of the study, a precise ratio, stable nutrient composition, and raw material source were selected with no differences between the same feed batches and no phytoestrogens or other exogenous substances (AIN-93G purified diet for feeding animals). RO pure water was selected as the drinking water for the animals.

### 2.2. Experimental Design

Timeline for in vivo studies: intracerebroventricular brain stereotaxic injection of the autophagy-specific inhibitor 3-methyladenine (3-MA, cat# 5142-23-4), the ER*α*-specific inhibitor methylpiperidino pyrazole (MPP, APExBIO, USA, cat# C3089) and the ER*β* specific inhibitor 4-(2-phenyl-5,7-bis(trifluoromethyl) pyrazolo [1,5-a] pyramidin-3-yl) phenol (PHTPP, APExBIO, USA, cat# B7144). ICA was given by intraperitoneal injection 20 min before HI (Shanghai Yuanye Bio-Technology Co., Ltd., China; HPLC ≥97%, cat# 489-32-7). The neonatal mice were nursed under rewarmed conditions for 1 h after the operation. After 1 h, the neonatal mice were put into a hypoxia box for continuous hypoxia exposure for 4 h. 2,3,5-Triphenyltetrazolium chloride (TTC) staining, brain water content, and western blotting experiments were performed on day 1 after HI. Terminal deoxynucleotidyl transferase dUTP nick-end labeling (TUNEL), Fluoro-Jade C (FJC), and tissue immunofluorescence studies were performed on day 3 after HI, and neurobehavioral testing was performed on days 1, 3, 5, and 7 after HI (Figure 1(b)).

#### 2.2.1. Experimental Design I

To evaluate the effect of ICA pretreatment on autophagy and the expression levels of the ER*α* and ER*β* proteins, P7 C57BL/6 neonatal mice were randomly divided into the following groups: sham, HI + Vehicle (solvent: 0.9% NaCl) and HI + ICA (10 mg/kg [17]), with 14 mice in each group. The expression levels of autophagy-related proteins as well as the proteins ER*α* and ER*β* were detected by immunofluorescence and western blot, respectively.

#### 2.2.2. Experimental Design II

To assess whether the neuroprotective effect of ICA pretreatment on apoptosis inhibition in HIBD neonatal mice was dependent on the mediation of autophagy, P7 C57BL/6 neonatal mice were randomly divided into the following groups: HI + ICA + Vehicle and HI + ICA+3-MA (2.48 *μ*g [55]), with 40 mice in each group. TTC staining was used to evaluate the volume of cerebral infarction in each pup, and cerebral edema injury was evaluated by brain water content. Behavioral tests were used to assess neurological function, and body weight was also measured. Immunofluorescence and western blotting were used to assess autophagy and apoptosis. FJC and TUNEL staining and caspase-3 evaluations were used to further assess apoptosis.

#### 2.2.3. Experimental Design III

To assess whether the neuroprotective effect produced by ICA pretreatment, which activated autophagy and thereby inhibited apoptosis, on HIBD neonatal mice was dependent on ER*α* mediation, P7 C57BL/6 neonatal mice were randomly divided into the following groups: HI + ICA + Vehicle and HI + ICA + MPP (50 nmol/mouse [56]), with 92 mice in each group. All experiments in Experimental design II were repeated, and the expression levels of the ER*α* and ER*β* proteins were detected.

#### 2.2.4. Experimental Design IV

To assess whether the neuroprotective effects produced by ICA pretreatment, which activated autophagy and thereby inhibited apoptosis in HIBD neonatal mice, were dependent on ER*β* mediation, P7 C57BL/6 neonatal mice were randomly divided into the following groups: HI + ICA + Vehicle and HI + ICA + PHTPP (50 nmol/mouse [56]), with 92 mice in each group. All experiments in Experimental design II were repeated, and the expression levels of the ER*α* and ER*β* proteins were detected.

### 2.3. Brain Stereotactic Localization and Lateral Ventricle Injection

Pups were anesthetized by continuous inhalation of isoflurane (3% for induction and 1.5% for maintenance [57]). The top of the head of each pup was sterilized with alcohol, and the head skin (approximately 0.3 cm) was incised along the midline of the head with a sterile blade to expose the bregma. A sterile cotton swab dipped in an appropriate amount of normal saline was used to gently wipe the surface of the calvaria, and a stereotaxic apparatus was used to locate and mark the lateral ventricle (according to the stereotaxic map of the mouse brain combined with the pup brain slice, the positioning coordinates of the pup were determined as 0.5 mm behind the bregma, 1 mm laterally and 1.5 mm deep). A drill was used to make a small hole at the marked position of the stereotaxic apparatus, and a Hamilton syringe (2 *μ*l liquid phase syringe, flat head) was used for injection (the injection volume was 2 *μ*l; the injection time was 5 min; the needle was retained for 5 min, and the needle was withdrawn over 5 min). After the injection, a proper amount of normal saline was applied to gently wipe the surface of the skull cap, and 3 M tissue glue was used to adhere the skin to the head. The neonatal mice were returned to the mother mice for warm nursing after the operation.

### 2.4. HIBD Model

The left common carotid artery (CCA) of P7 C57BL/6 newborn mice was ligated using the modified Rice-Vannucci [58, 59] modeling method. In the sham group, only the CCA was exposed, and HI treatment was not administered. The neonatal mice were fixed on the operating table on their back, and continuous isoflurane inhalation anesthesia (3% for induction and 1.5% for maintenance) was performed on the neonatal mice using an anesthesia mask. The neck of each neonatal mouse was wiped with an alcohol-containing cotton ball, and a median incision (3 mm) was made in the neck with surgical scissors. A pulsatile CCA was found in the deep carotid sheath on the left side of the trachea, dissociated and permanently burned out with an electric pen. The wounds were sutured after the operation. After the neonatal mice regained consciousness, they were returned to the mother mice for warm nursing for 1 h. The neonatal mice were then placed in a 37°C hypoxia chamber (8% O_2_+92% N_2_, gas flow rate of 2 L/min) for 4 h of continuous hypoxia. After hypoxia, the neonatal mice were returned to the side of the female mice for lactation, and the model was completed.

### 2.5. Neurobehavioral Assessments

On the 1st, 3rd, 5th, and 7th days after HI, two fixed researchers carried out the righting reflex, negative geotaxis, and grip tests on C57BL/6 newborn mice both 8 : 00 AM and 8 : 00 PM without clear grouping and recorded the weights of the pups [60]. All behavioral experiments were conducted in a quiet environment, with few people stirring and gentle movements.

#### 2.5.1. Righting Reflex

Neonatal mice were placed on a plane operating table in the supine position, and one hand was gently pressed on the body. Then, the hand was released, and the time to plane righting from the supine position was recorded. The test was repeated three times for each pup, and if the pup did not return to its original position within 60 s after being turned over, the time was recorded as 60 s.

#### 2.5.2. Negative Geotaxis

A plane with a changeable angle was prepared in advance. Each pup was placed on the plane, with the head end of the pup facing in the tilt direction of the plane. When the pup was relaxed and still, the plane was quickly tilted by 45°, and the time required for the pup to make a 180° turn from the rest to the head and body was recorded. The test was repeated 3 times for each pup. If the pup did not turn its head and body within 60 s, the time was recorded as 60 s.

#### 2.5.3. Grip Test

A test box was prepared as the experimental device, which was 50 cm long, 50 cm wide, and 15 cm high. There was a wire in the middle of the box (diameter of approximately 1.5 mm), and cork chips were laid underneath. After the pup grasped the metal wire with their bilateral front feet, the experimenter released their hands, and the time at which the pup released the metal wire was recorded. The test was repeated 3 times for each pup.

### 2.6. TTC Staining

TTC (Sigma–Aldrich, Germany, CAT# G3005) staining was used to assess infarct volume in neonatal mice. Twenty-four hours after HIBD, the neonatal mice were anesthetized with isoflurane, and brain tissue samples were taken, put in cell culture dish at -20°C for 13 min. The brain tissue was removed and cut into four average brain slices on the coronal plane of the brain. The brain sections were placed in 1% TTC dye solution (1 ml of ddH_2_O+1 ml of 2% TTC dye solution) and stained in the dark at 37°C for 20 min. During the staining, the brain sections were frequently turned to make them uniformly in contact with the dye solution. After staining, the brain sections were fixed with 4% paraformaldehyde (PFA), and the brain sections were scanned by the scanner the next day. Normal brain tissue is red, while brain tissue with HI damage is white. Percentage of cerebral infarct volume = [(contralateral hemisphere − ipsilateral uninfarcted area)/contralateral hemisphere × 2] × 100% [61].

### 2.7. Brain Water Content

Twenty-four hours after HIBD, the neonatal mice were anesthetized with isoflurane, and brain tissue samples were taken to measure the wet weights of the left and right cerebral hemispheres. Subsequently, the cerebral hemispheres were dried in an oven at 106°C for 24 h for dry weight measurement. Percentage of brain water content = (wet weight − dry weight)/wet weight × 100% [60].

### 2.8. Preparation of Tissue Paraffin Sections

Twenty-four hours after HIBD, the neonatal mice were anesthetized by continuous inhalation of isoflurane (3% for induction and 1.5% for maintenance). The neonatal mice were given cardiac perfusion with ice-cold normal saline and 4% PFA; brain tissue was extracted and fixed in 4% PFA at 4°C for 24 h. After 24 h, the fixed brain tissues were rinsed with fine running water for 16 h and placed in an automatic dehydrator for dehydration. The dehydration step was carried out according to the following protocol: 70% ethanol (1 h)-80% ethanol (1 h)-90% ethanol (1 h)-95% ethanol I (1 h)-95% ethanol II (1 h)-100% ethanol I (1 h)-100% ethanol II (1 h)-xylene I (1 h)-xylene II (30 min)-wax I (low-melting point wax, at 48-50°C, 30 min)-wax II (52-54°C medium melting point wax, 30 min)-wax IIIIII (54-56°C high melting point wax, 30 min). Then, the tissue was embedded in paraffin. Sections were made using a microtome (section thickness of 4 *μ*m). Finally, the brain tissue slices were placed in a 37°C oven for 48 h to complete preparation.

### 2.9. TUNEL Staining of Tissue

A TUNEL Apoptosis Detection Kit (Fluorescence) (Wanleibio, China, cat# WLA030A) was used to detect apoptotic cells. Brain tissue sections were selected and placed in xylene I and II for 15 min each. Then, the cells were placed in 100% alcohol I, alcohol II, and 95%, 90%, 80%, 70%, and 50% alcohol for 5 min each before gently washing with ddH_2_O 3 times. The brain tissue sections were placed in 0.01 mol/L sodium citrate buffer for microwave antigen repair for 15 min. Next, 50 *μ*l of 3% H_2_O_2_ was added dropwise to each brain tissue section. Sections were placed in the dark for 10 min and then washed with PBS (3 × 5 min). Then, 50 *μ*l of TUNEL reaction buffer (1 part 10 × enzyme reagent + 9 parts1 × labeling substrate) was added dropwise to each tissue section before incubation in a wet box at 37°C for 90 min in the dark and washing with PBS (3 times × 5 min). Finally, 10 *μ*l of antifluorescence quencher (including DAPI) was added dropwise to the brain tissue before it was mounted, fixed, and imaged with a fluorescence microscope.

### 2.10. FJC Staining

FJC staining (Biosensis, USA, cat# TR-100-FJT) was used to mark degenerated neurons. Brain tissue sections were selected and placed in xylene I and II for 15 min each. Then, the cells were placed in 100% alcohol I, alcohol II, and 95%, 90%, 80%, 70%, and 50% alcohol for 5 min each before gently washing with ddH_2_O 3 times. A mixture of ddH_2_O and potassium permanganate solution (9 : 1) was added dropwise to the brain tissue sections (200 *μ*l per brain sample) before incubation for 10 min and two washes (2 min each) with ddH_2_O. Solutions of ddH_2_O, FJC, and DAPI were mixed at a ratio of 1 : 1 : 1, dropped onto the brain tissue sections and incubated in the dark for 20 min (200 *μ*l for each brain sample). Sections were gently washed with ddH_2_O three times for 1 min each time. The brain sections were dried in an oven at 56°C for 5 min and made transparent by being placed in a xylene solution. After the excess water around the brain tissues was absorbed with filter paper, 10 *μ*l of antifluorescence quencher was applied to the brain tissues dropwise; the sections were sealed and fixed, and pictures were taken with a fluorescence microscope.

### 2.11. Tissue Immunofluorescence Staining

Brain tissue sections were selected and placed in xylene I and II for 15 min each. Then, the cells were placed in 100% alcohol I, alcohol II, and 95%, 90%, 80%, 70%, and 50% alcohol for 5 min each and gently washed with ddH_2_O 3 times. The brain tissue sections were placed in 0.01 mol/L sodium citrate buffer for microwave antigen repair for 20 min and then washed three times with phosphate-buffered saline with Tween 20 (PBST). The tissues were blocked with Quick Block™ immunostaining blocking solution (Beyotime Institute of Biotechnology, China, cat# P0260) for 20 min and then washed three times with PBST. The primary antibody was added according to the antibody instructions concentration range using phosphate-buffered solution (PBS): ER*α* (1 : 200, Proteintech, USA, cat# 21244-1-AP), ER*β* (1 : 100, Proteintech, USA, cat# 14007-1-AP), Beclin1 (1 : 200, Abcam, USA, cat# ab217179), LC3 (1 : 200, Abcam, USA, cat# ab192890), recombinant sequestosome 1 (SQSTM1)/p62 (1 : 200, Abcam, USA, cat# ab91526), tumor suppressor gene (p53) (1 : 1600, Proteintech, USA, cat# 60283-2-lg), 53 upregulated modulator of apoptosis 9 (PUMA) (1 : 100, Multisciences, China, cat# ab40081), Bcl-2-associated X (Bax) (1 : 200, Proteintech, USA, cat# 60267-1-lg), Bcl-2 (1 : 200, Proteintech, USA, cat# 26593-1-AP), caspase-3 (1 : 50, Proteintech, USA, cat# 19677-1-AP), and cleaved caspase-3 (1 : 200, Affinity, USA, cat# AF7022). Then, 25 *μ*l of the prepared primary antibody was added dropwise to each brain tissue sample for incubation in a 4°C refrigerator for 16 h. The samples were rewarmed for 30 min the next day; the primary antibody was discarded, and the tissue was washed three times with PBST. Next, 50 *μ*l of DyLight 488-labeled goat anti-rabbit or anti-mouse fluorescence secondary antibody (1 : 360, Earthox, USA, cat# E032210-01) or DyLight 594-labeled goat anti-rabbit fluorescent secondary antibody (1 : 360, Earthox, USA, cat# E032420-01) was dropwise added to each brain tissue section, and the sections incubated in the dark at room temperature for 2 h. The sections were washed 3 times with PBST (5 min each). Then, 10 *μ*l of antifluorescence quencher (including DAPI) was added dropwise onto the brain tissue samples. Sections were mounted and fixed, and pictures were taken with a fluorescence microscope.

### 2.12. Caspase 3 Activity Detection

The procedures were performed according to the experimental protocol of the caspase-3 activity detection kit (BestBio, China, CAT # BB-4106). Tissue proteins were extracted, and bicinchoninic acid (BCA) protein quantification was performed. Then, 50 *μ*l of quantified protein lysis supernatant was added to the sample wells of a 96-well plate, and 40 *μ*l of prepared detection buffer (10 *μ*l of DL-dithiothreitol (DTT) in every 1 ml of buffer) and 10 *μ*l of caspase-3 substrate were added. After the reagents were fully mixed, the samples were incubated in a 37°C incubator for 16 h in the dark. A microplate reader was used to measure the absorbance of the samples at A405 nm, and the relative activity of caspase-3 was calculated according to the absorbance of apoptotic cells/the absorbance of control cells.

### 2.13. Protein Extraction and Western Blot Detection

*Tissue protein extraction*: twenty-four hours after HIBD initiation in neonatal mice, isoflurane was used to induce anesthesia, and brain tissue samples were extracted. RIPA lysis buffer was added to lyse brain tissue samples before being ground using a grinder and an ultrasonic cell grinder. The ground brain tissue samples were placed in a centrifuge at 4°C for centrifugation at 12000 rpm for 15 min, and the supernatant was extracted and quantified by BCA.

*Western blot detection*: first, an 8%, 10%, or 12% separating gel and 5% of the concentrated gel was prepared (Beyotime Institute of Biotechnology, China, cat# P0012A).

*Loading*: the loading volume of tissue protein was 15 *μ*l. Electrophoresis was carried out at 80 V for 60 min. Then, the proteins were transferred to a membrane (300 mA, 100 min) and blocked with 5% skimmed milk powder for 2 h. The primary antibodies were added for incubation at 4°C for 16 h: ER*α* (1 : 1000, Proteintech, USA, cat# 21244-1-AP), ER*β* (1 : 2000, Proteintech, USA, cat# 14007-1-AP), Beclin1 (1 : 1000, Abcam, USA, cat# ab217179), LC3 (1 : 1000, Abcam, USA, cat# ab192890), p62 (1 : 1000, Abcam, USA, cat# ab91526), p53 (1 : 1000, Proteintech, USA, cat# 60283-2-lg), PUMA (1 : 1000, Multisciences, China, cat# ab40081), Bax (1 : 5000, Proteintech, USA, cat# 60267-1-lg), Bcl-2 (1 : 1000, Proteintech, USA, cat# 26593-1-AP), caspase-3 (1 : 500, Proteintech, USA, cat# 19677-1-AP), and cleaved caspase-3 (1 : 500, Affinity, USA, cat# AF7022). Then, goat anti-rabbit secondary antibody or goat anti-mouse secondary antibody was added for 1 h of incubation at room temperature, and the membranes were photographed after developing.

### 2.14. Statistical Analysis

All experiments were repeated at least 3 times, and the experimental data are expressed as the mean ± SEM. SPSS 21.0 and GraphPad Prism software (version 8.0, USA) were used for statistical analysis of the experimental data. One-way ANOVA was used for analysis of the differences between multiple groups, and then post hoc testing was performed with Tukey's or Student-Newman–Keuls multiple comparisons tests. Differences between two groups were compared using Student's *t* test. *P* < 0.05 indicated a statistically significant difference.

## 3. Results

### 3.1. ICA Pretreatment Significantly Promoted Autophagy in HIBD Neonatal Mice

**Figure 2:**
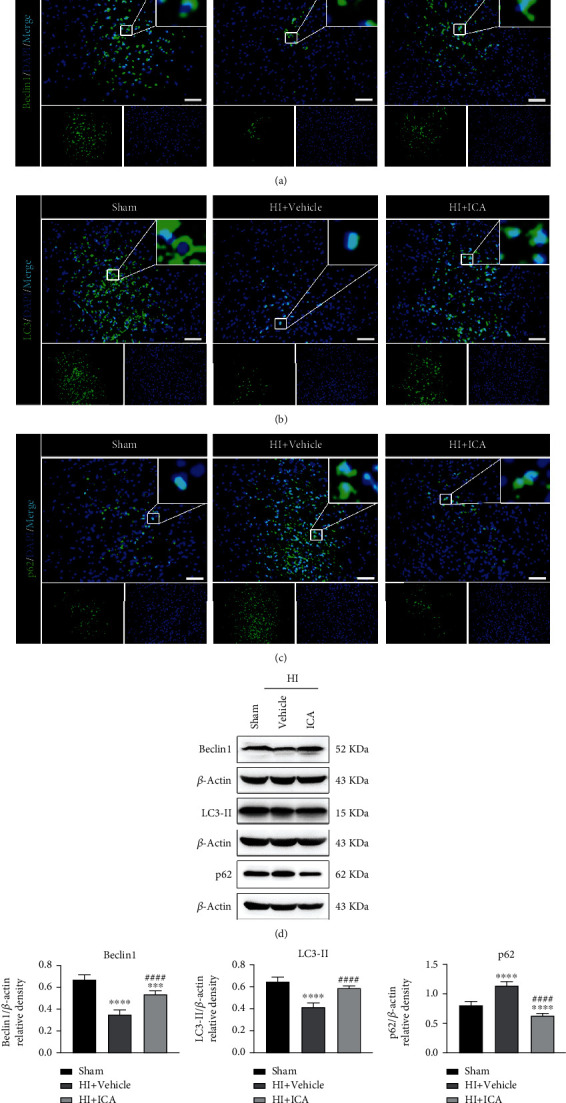
Effect of ICA pretreatment on autophagy in HIBD newborn mice. The expression levels of (a) Beclin1, (b) LC3, and (c) p62 in the cerebral ischemic penumbra (IP, the small black box in the upper left brain slice; the neurons in this area are in a state of electrical failure, and the focus on saving these cells is the key and hot spot for the treatment of HIBD) of neonatal HIBD mice pretreated with ICA were detected by immunofluorescence, *n* = 8 mice in each group. Bar = 100 *μ*m. (d) Representative western blot images and (e) quantitative analysis. ^∗∗^*P* < 0.01, ^∗∗∗^*P* < 0.001, and ^∗∗∗∗^*P* < 0.0001 compared to the sham group,^##^*P* < 0.01 and ^####^*P* < 0.0001 compared to the HI + Vehicle group. Data are presented as the mean ± SDs, *n* = 6 mice in each group.

To explore whether ICA pretreatment inhibits apoptosis by regulating autophagy in HIBD newborn mice, we first clarified the effect of ICA pretreatment on the level of autophagy in HIBD newborn mice. The immunofluorescence staining results (Figures 2(a)–2(c)) showed a significant decrease in the numbers of Beclin1- (Figure 2(a)) and LC3-positive cells (Figures 2(a) and 2(b)) significant increase in the number of p62-positive cells (Figure 2(c)), and ICA pretreatment significantly reversed these findings after HIBD induction in neonatal mice compared with the sham group. The western blot results were consistent with the immunofluorescence results (Figures 2(d)–2(g)). Thus, ICA pretreatment significantly promoted autophagy in the brains of HIBD newborn mice.

To further verify the above results, we constructed an oxygen and glucose deprivation (OGD) model by culturing HT22 cells in vitro. First, preliminary experiments showed that the optimal hypoxia time of OGD was 4 h, and the optimal dose of ICA was 8 *μ*mol/L (Supplementary material 1), which further verified and supported the above in vivo experimental results (Supplementary material 2). These results suggested that the neuroprotective mechanism of ICA pretreatment on HIBD in neonatal mice might be related to the activation of autophagy.

### 3.2. The Autophagy Inhibitor 3-MA Reversed the Neuroprotective Effect of ICA Pretreatment in HIBD Newborn Mice

**Figure 3:**
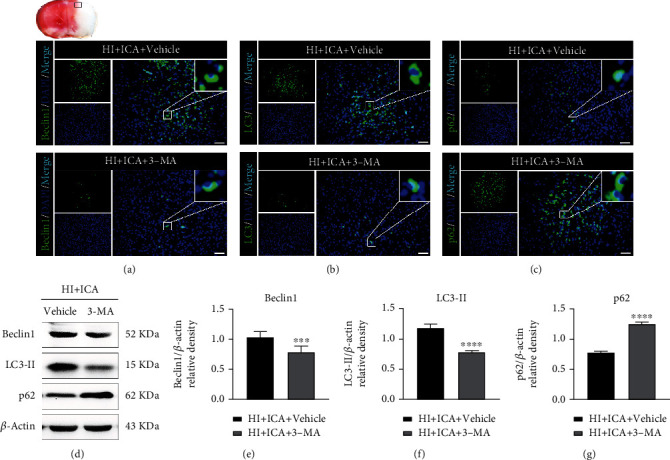
Effects of the autophagy inhibitor 3-MA on autophagy-related proteins in HIBD neonatal mice pretreated with ICA. Immunofluorescence detection of the expression of (a) Beclin1, (b) LC3, and (c) p62 in the ischemic penumbra (small black box in the upper left brain slice) of 3-MA-treated HIBD neonatal mice, *n* = 8 mice in each group. Bar = 100 *μ*m. (d) Representative western blot images and (e–g) quantitative analysis. ^∗∗∗^*P* < 0.001, and ^∗∗∗∗^*P* < 0.0001 compared to the HI + ICA + Vehicle group. Data are presented as the mean ± SDs, *n* = 6 mice in each group.

We further explored whether the neuroprotective effect of ICA on HIBD newborn mice depends on the increase in autophagy level mentioned above. In this study, the autophagy inhibitor 3-MA was used to detect its effect on the neurological function of HIBD newborn mice pretreated with ICA. The in vivo immunofluorescence staining results (Figures 3(a)–3(c)) showed that the numbers of Beclin1- (Figure 3(a)) and LC3-positive cells (Figure 3(b)) significantly decreased, and the number of p62-positive cells (Figure 3(c)) significantly increased in 3-MA-treated pups when compared with those in the HI + ICA + Vehicle group. The western blot results were consistent with the immunofluorescence results (Figures 3(d)–3(g)). Similar results were achieved after in vitro cellular validation, supporting our in vivo findings (Supplementary material 3). The above experimental results showed that the autophagy inhibitor 3-MA significantly inhibited autophagy in HIBD newborn mice pretreated with ICA.

**Figure 4:**
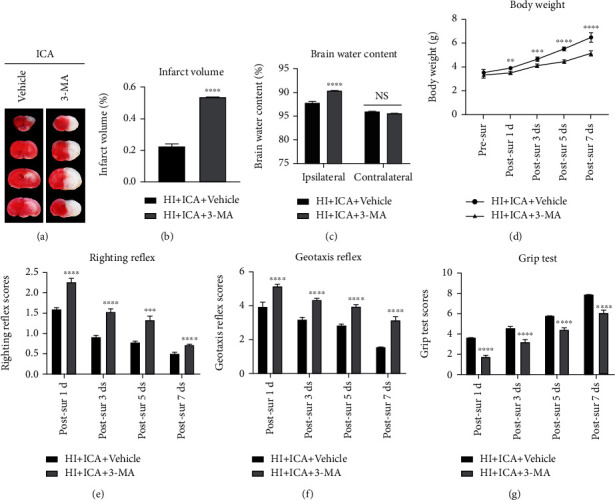
Effects of the autophagy inhibitor 3-MA on cerebral infarction volume, cerebral water content, and neurological function in HIBD newborn mice. (a) TTC staining photos of brain tissue sections, *n* = 6 in each group. (b) Quantitative analysis of TTC staining showing cerebral infarct volume. (c) Quantitative analysis of the brain water content in neonatal mice with HIBD, *n* = 6 in each group. (d) Quantitative analysis of body weight measurements, (e) positive righting reflex, (f) negative geotaxis reflex, and (g) grip tests on days 1, 3, 5, and 7 after HIBD in neonatal mice, *n* = 8 mice in each group. ^∗∗^*P* < 0.01, ^∗∗∗^*P* < 0.001, and ^∗∗∗∗^*P* < 0.0001 compared to the HI + ICA + Vehicle group, NS = no significant difference. Data are expressed as the *mean* ± *SDs*.

To further test the effect of the autophagy inhibitor 3-MA on the neuroprotective effect of ICA pretreatment on newborn mice with HIBD, TTC staining was carried out, and the results showed a significant increase in cerebral infarct volume in the 3-MA-treated pups compared with those in the HI + ICA + Vehicle group (Figures 4(a) and 4(b)). Moreover, pups administered 3-MA displayed significantly increased brain water contents than the pups in the HI + ICA + Vehicle group (Figure 4(c)). Body weight measurements showed that the pups treated with 3-MA lost significantly more weight at 1, 3, 5, and 7 d postsurgery than the pups in the HI + ICA + Vehicle group (Figure 4(d)). The results of the righting reflex test (Figure 4(e)), negative geotaxis test (Figure 4(f)), and grip test (Figure 4(g)) all showed that 3-MA treatment significantly aggravated the neurological impairment in the neonatal mice on the 1st, 3rd, 5th, and 7th d after surgery. We, therefore, concluded that inhibiting autophagy by 3-MA treatment reverses the neuroprotective effect of ICA pretreatment on HIBD newborn mice to aggravate HIBD. These results suggested that autophagy activation might play an important role in the neuroprotection of neonatal mice with HIBD.

### 3.3. The Autophagy Inhibitor 3-MA Reversed the Inhibitory Effect of ICA Pretreatment on Apoptosis in HIBD Newborn Mice

**Figure 5:**
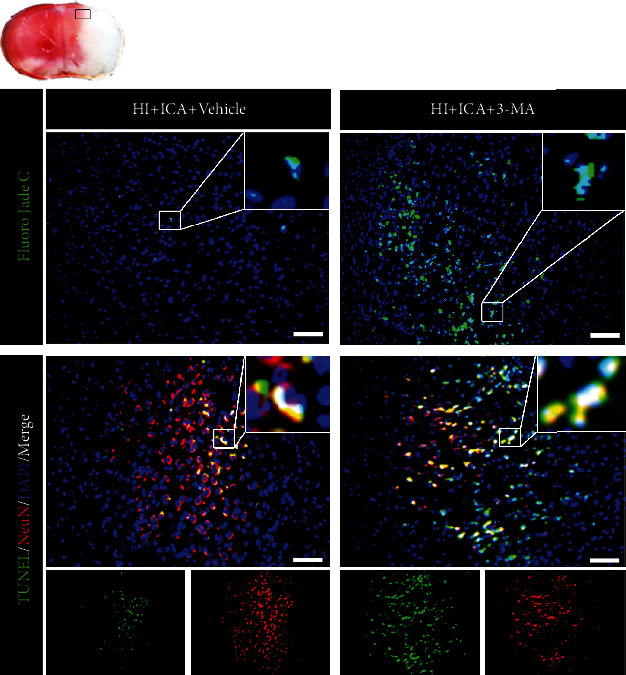
Effect of the autophagy inhibitor 3-MA on the level of apoptosis in HIBD neonatal mice pretreated with ICA. The numbers of TUNEL-positive cells (green fluorescence in the lower panel), NeuN-positive cells (red fluorescence), and FJC-positive neurons (green fluorescence in the upper panel) in the cerebral ischemic penumbra (small black box in the upper left brain slice) in 3-MA-treated HIBD newborn mice detected by tissue immunofluorescence, *n* = 8 mice in each group. Bar = 100 *μ*m.

**Figure 6:**
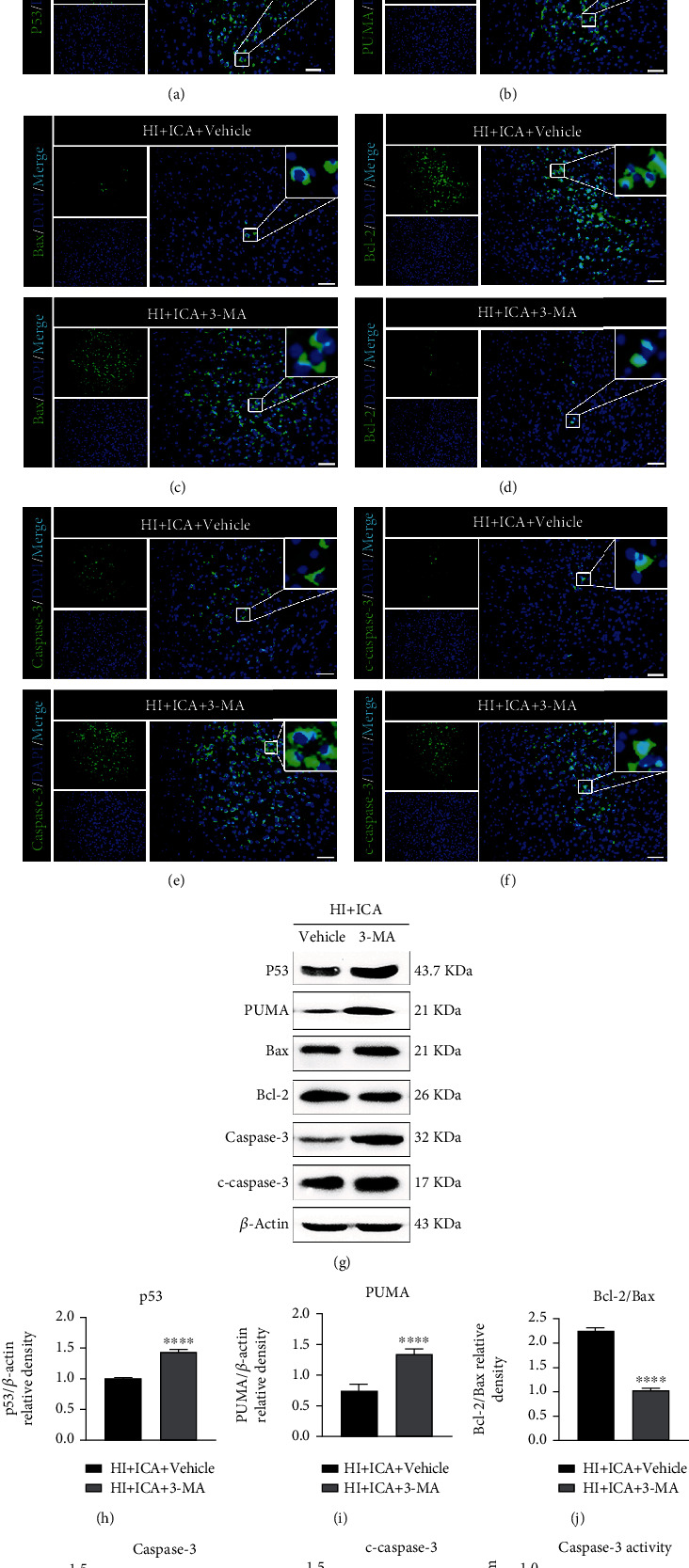
Effects of the autophagy inhibitor 3-MA on apoptosis-related proteins in HIBD neonatal mice pretreated with ICA. The expression levels of (a) p53, (b) PUMA, (c) Bax, (d) Bcl-2, (e) caspase-3, and (f) cleaved caspase-3 in the cerebral ischemic penumbra (small black box in the upper left brain section) in 3-MA-treated HIBD newborn mice detected by tissue immunofluorescence, *n* = 8 mice in each group. Bar = 100 *μ*m. (g) Representative western blot images and (h–l) quantitative analysis. (m) Quantitative analysis results of caspase-3 activity detection. ^∗∗∗^*P* < 0.001, and ^∗∗∗∗^*P* < 0.0001 compared to the HI + ICA + Vehicle group. Data are presented as the mean ± SDs, *n* = 6 mice in each group.

**Figure 7:**
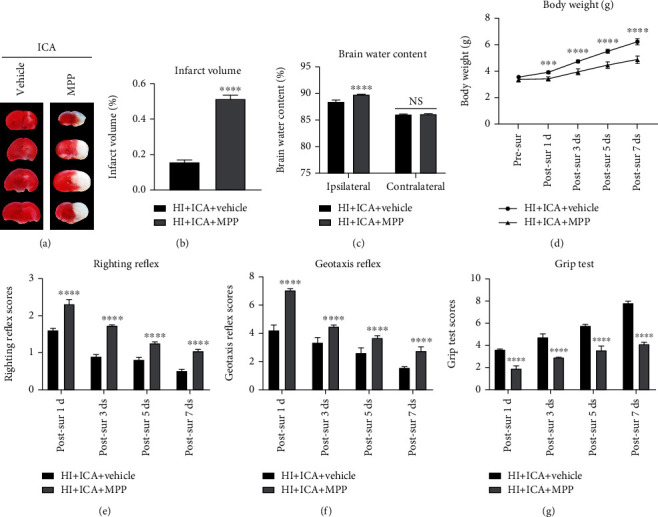
Effects of the ER*α* inhibitor MPP on cerebral infarction volume, cerebral water content, and neurological function in HIBD newborn mice. (a) TTC staining photos of brain tissue sections, *n* = 6 mice in each group. (b) Quantitative analysis of cerebral infarct volume shown by TTC staining. (c) Quantitative analysis of brain water content in HIBD pups, *n* = 6 mice in each group. (d) Quantitative analyses of body weight, (e) righting reflex, (f) negative geotaxis, and (g) grip tests on days 1, 3, 5, and 7 after HIBD in neonatal mice, *n* = 8 mice in each group. ^∗∗∗^*P* < 0.001 and ^∗∗∗∗^*P* < 0.0001 compared with the HI + ICA + Vehicle group, NS = no significant difference. Data are presented as the mean ± SDs.

Previous studies by our group showed that ICA pretreatment inhibits apoptosis to exert neuroprotective effects on HIBD newborn mice [17]. However, is the inhibition of apoptosis caused by ICA related to autophagy in HIBD newborn mice? TUNEL and FJC staining (Figure 5) showed that compared with the HI + ICA + Vehicle group, the number of TUNEL-positive cells and FJC-positive neurons in the 3-MA-treated pups were significantly increased. Immunofluorescence staining of tissues (Figures 6(a)–6(f)) showed that the numbers of p53- (Figure 6(a)), PUMA- (Figure 6(b)), Bax- (Figure 6(c)), caspase-3- (Figure 6(e)), and cleaved caspase-3-positive cells (Figure 7(f)) were significantly higher and the number of Bcl-2-positive cells (Figure 7(d)) was significantly lower in the 3-MA-treated pups than in the HI + ICA + Vehicle group. The western blot results were consistent with the immunofluorescence results (Figures 6(g)–6(l)). Caspase-3 activity (Figure 6(m)) increased significantly in 3-MA-treated pups. Similar results were achieved with cells in vitro, supporting our findings in vivo (Supplementary material 4). The above experimental results indicated inhibiting autophagy significantly promoted the apoptosis in HIBD newborn mice pretreated with ICA, and the neuroprotective effect of ICA apoptosis inhibition in HIBD newborn mice might be mediated by the activation of autophagy.

### 3.4. ICA Pretreatment Significantly Upregulated ER*α* and ER*β* Levels in HIBD Newborn Mice

**Figure 8:**
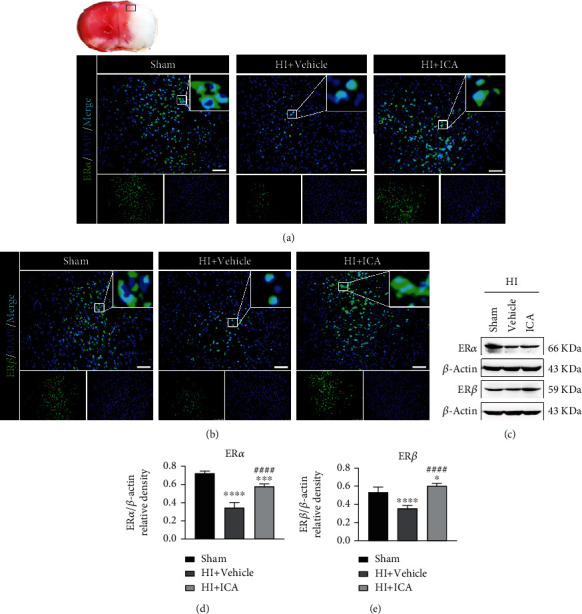
Effect of ICA pretreatment on ER*α* and ER*β* levels in neonatal mice with HIBD. Immunofluorescence was used to detect the expression of (a) ER*α* and (b) ER*β* in the ischemic penumbra of HIBD neonatal mice pretreated with ICA (the small black frame area in the upper left corner of the brain slice), *n* = 8 mice in each group. Bar = 100 *μ*m. (c) Representative western blot images and (d, e) quantitative analysis. ^∗∗∗^*P* < 0.001, and ^∗∗∗∗^*P* < 0.0001 compared to the sham group, ^####^*P* < 0.0001 compared to the HI + Vehicle group. Data are presented as the mean ± SDs, *n* = 6 mice in each group.

To investigate whether ER*α* and ER*β* are involved in the mechanism by which ICA pretreatment is neuroprotective in HIBD neonatal mice, we examined the expression levels of the ER*α* and ER*β* proteins. Immunofluorescence staining (Figures 8(a) and 8(b)) showed a significant reduction in the number of ER*α*- and ER*β*-positive cells after HIBD in pups when compared to the sham group; a result that was significantly reversed by ICA pretreatment. The western blot results were consistent with the immunofluorescence data (Figures 8(c)–8(e)). Similar results were achieved with cells in vitro, supporting our findings in vivo (Supplementary material 5). We can therefore conclude that ICA pretreatment significantly increased the expression levels of ER*α* and ER*β* after HIBD.

### 3.5. The ER*α* Inhibitor MPP Reversed the Neuroprotective Effects of ICA Pretreatment on HIBD Newborn Mice

**Figure 9:**
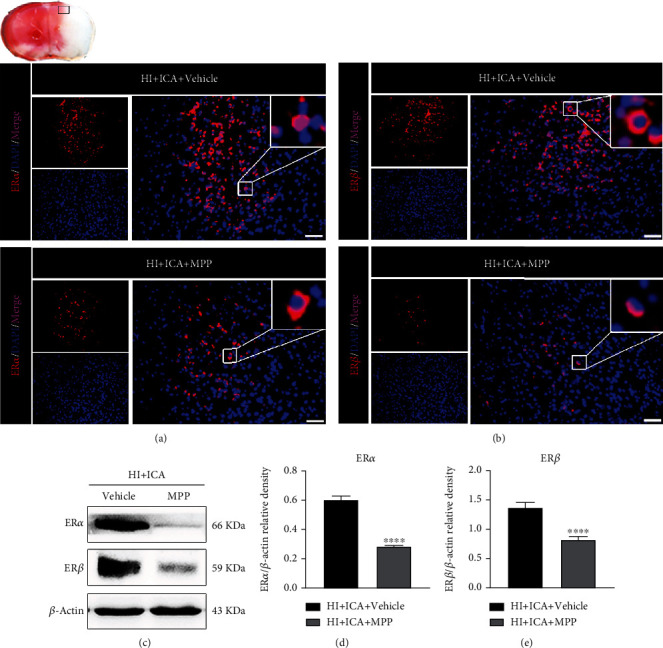
Effects of the ER*α* inhibitor MPP on the levels of ER*α* and ER*β* in HIBD newborn mice pretreated with ICA. The expression of (a) ER*α* and (b) ER*β* in the cerebral ischemic penumbra (small black box in the upper left brain section) of MPP-treated HIBD newborn mice detected by immunofluorescence, *n* = 8 mice in each group. Bar = 100 *μ*m.

We next explored whether the neuroprotective effect of ICA on HIBD newborn mice depends on the increase in the ER*α* level in the above cells. However, we first needed to verify the inhibitory effect of MPP on ER*α* and explore the influence of ER*α* inhibition on ER*β* expression in HIBD newborn mice pretreated with ICA. The immunofluorescence staining results (Figures 9(a) and 9(b)) showed that the number of ER*α*- and ER*β*-positive cells in the group of MPP-treated pups was significantly reduced compared with that in the HI + ICA + Vehicle group. The western blot results were consistent with the immunofluorescence data (Figures 9(c)–9(e)). In vitro, the optimal inhibitory concentration of MPP was determined through preliminary experiments to be 1 *μ*mol/L (Supplementary material 6), and the in vitro results were similar to those above (Supplementary material 7), thus supporting and verifying the findings obtained in vivo. The above experimental data showed that MPP treatment effectively inhibited the expression of ER*α* in ICA-pretreated HIBD neonatal mice and produced a significant inhibitory effect on ER*β*.

We further explored the role of the ER*α* inhibitor MPP on neuroprotection in ICA-pretreated HIBD neonatal mice. The results of the TTC (Figures 7(a) and 7(b)), brain water content (Figure 7(c)), body weight measurement (Figure 7(d)), and neurobehavioral experiments (Figures 7(e)–7(g)) were consistent with the effects observed with the autophagy inhibitor 3-MA. We thus concluded that MPP treatment not only reversed the neuroprotective effects of ICA pretreatment on HIBD neonatal mice but also exacerbated HIBD. These results suggested that ER*α* activation might play an important role in neuroprotection in HIBD in neonatal mice.

### 3.6. The ER*α* Inhibitor MPP Reversed the Promotion of Autophagy Resulting from ICA Pretreatment in HIBD Newborn Mice

**Figure 10:**
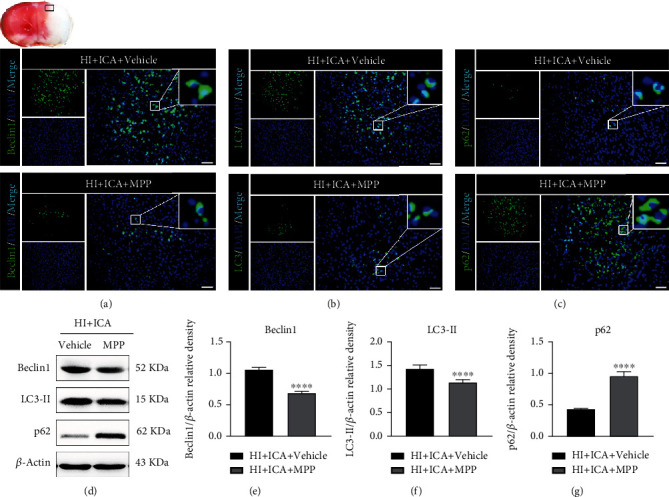
Effects of the ER*α* inhibitor MPP on autophagy-related proteins in HIBD newborn mice pretreated with ICA. The expression of (a) Beclin1, (b) LC3, and (c) p62 in the cerebral ischemic penumbra (black box of the brain slice in the upper left corner) in neonatal HIBD mice treated with MPP detected by fluorescence, *n* = 8 mice in each group. Bar = 100 *μ*m. (d) Representative western blot images and (e–g) quantitative analysis. ^∗∗∗∗^*P* < 0.0001 compared to the HI + ICA + Vehicle group. Data are presented as the mean ± SDs, *n* = 6 mice in each group.

We then examined the effect of the ER*α* inhibitor MPP on the level of autophagy in ICA-pretreated HIBD neonatal mice. Immunofluorescence staining (Figures 10(a)–10(c)) showed that compared with the HI + ICA + Vehicle group, the number of Beclin1- (Figure 10(a)) and LC3-positive cells (Figure 10(b)) in MPP-treated pups was significantly reduced, and the number of p62-positive cells (Figure 10(c)) was significantly increased. The western blot results were consistent with the immunofluorescence data (Figures 10(d)–10(g)). Similar results were achieved with cells in vitro, supporting our findings in vivo (Supplementary material 8). The above experimental results indicated that MPP treatment significantly reversed the effects by which ICA pretreatment promotes autophagy in HIBD newborn mice and OGD-injured HT22 cells and inhibited autophagy.

### 3.7. The ER*α* Inhibitor MPP Reversed the Inhibition of Apoptosis Resulting from ICA Pretreatment in HIBD Newborn Mice

**Figure 11:**
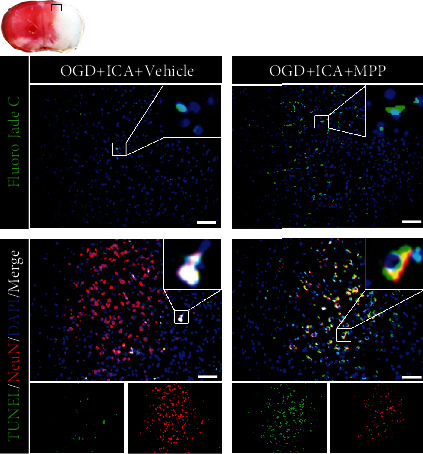
Effect of the ER*α* inhibitor MPP on apoptosis in HIBD newborn mice pretreated with ICA. The numbers of TUNEL-positive cells (green fluorescence in the lower panel), NeuN-positive cells (red fluorescence), and FJC-positive neurons (green fluorescence in the upper panel) in the cerebral ischemic penumbra (small black box in the upper left brain section) in MPP-treated HIBD newborn mice detected by tissue immunofluorescence, *n* = 8 mice in each group. Bar = 100 *μ*m.

**Figure 12:**
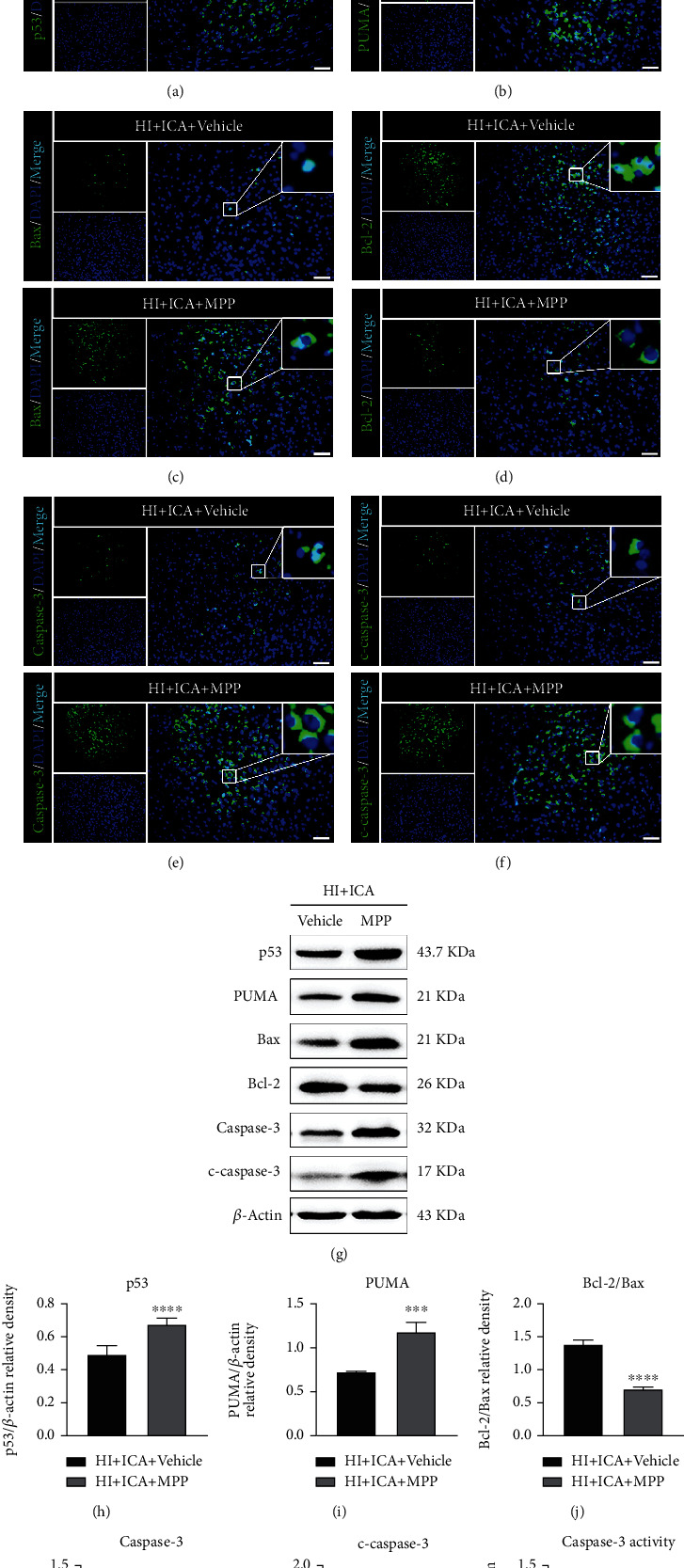
The ER*α* inhibitor MPP inhibits the expression of apoptosis-related proteins in HIBD neonatal mice pretreated with ICA. The expression levels of (a) p53, (b) PUMA, (c) Bax, (d) Bcl-2, (e) caspase-3, and (f) cleaved caspase-3 in the cerebral ischemic penumbra (small black box in the upper left brain section) in MPP-treated HIBD newborn mice detected by tissue immunofluorescence, *n* = 8 mice in each group. Bar = 100 *μ*m. (g) Representative western blot images and (h–l) quantitative analysis. (m) Quantitative analysis of the detected caspase-3 activity. ^∗∗^*P* < 0.01, ^∗∗∗^*P* < 0.001, and ^∗∗∗∗^*P* < 0.0001 compared to the HI + ICA + Vehicle group. Data are presented as the mean ± SDs, *n* = 6 mice in each group.

Further, we examined the effect of the ER*α* inhibitor MPP on the level of apoptosis in ICA-pretreated HIBD neonatal mice. TUNEL and FJC staining showed that compared with the HI + ICA + Vehicle group, the numbers of TUNEL-positive cells and FJC-positive neurons in MPP-treated pups were significantly increased (Figure 11). Tissue immunofluorescence staining (Figures 12(a)–12(f)) showed that, compared with the HI + ICA + Vehicle group, after MPP treatment, the number of p53- (Figure 12(a)), PUMA- (Figure 12(b)), Bax- (Figure 12(c)), caspase-3- (Figure 12(e)), and cleaved caspase-3-positive cells (Figure 12(f)) increased significantly. Additionally, the number of Bcl-2-positive cells (Figure 12(d)) was significantly reduced. The western blot results were consistent with the immunofluorescence data (Figures 12(g)–12(m)). Moreover, caspase-3 activity (Figure 12(m)) was significantly increased in MPP-treated pups. Similar results were achieved with cells in vitro, supporting our findings in vivo (Supplementary material 9). The above results indicated inhibiting the ER*α* protein with the ER*α* inhibitor MPP significantly promoted apoptosis in HIBD neonatal mice pretreated with ICA.

### 3.8. The ER*β* Inhibitor PHTPP Reversed the Neuroprotective Effects Resulting from ICA Pretreatment in HIBD Newborn Mice

**Figure 13:**
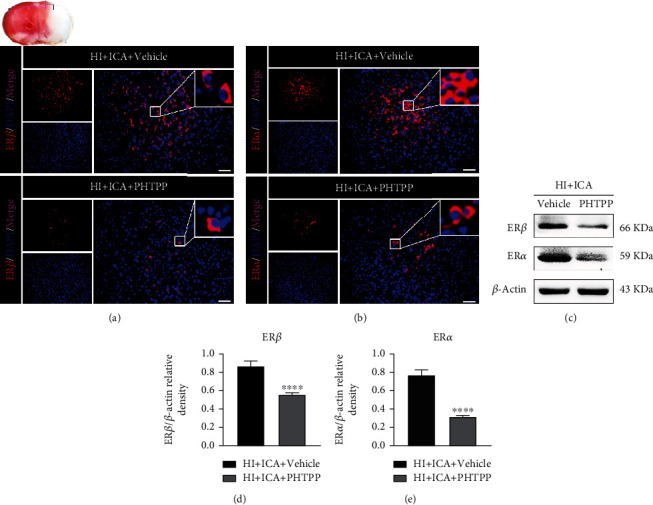
Effects of the ER*β* inhibitor PHTPP on the ER*β* and ER*α* levels in HIBD newborn mice pretreated with ICA. The expression levels of (a) ER*β* and (b) ER*α* in the cerebral ischemic penumbra (small black box in the upper left brain section) in PHTPP-treated HIBD newborn mice detected by immunofluorescence, *n* = 8 mice in each group. Bar = 100 *μ*m. (a) Representative western blot images and (d, e) quantitative analysis. ^∗∗∗∗^*P* < 0.0001 compared to the HI + ICA + Vehicle group. Data are presented as the mean ± SDs, *n* = 6 mice in each group.

In this study, ICA pretreatment upregulated both ER*α* and ER*β* levels in HIBD newborn mice. We then asked, does the neuroprotective effect of ICA on HIBD newborn mice depend on the increase in ER*β* levels? First, it was necessary to verify the inhibitory effect of the ER*β* inhibitor PHTPP on ER*β* and the effect of ER*β* inhibition on ER*α* expression levels in ICA-pretreated HIBD neonatal mice. Immunofluorescence staining (Figures 13(a) and 13(b)) showed a significant reduction in the number of ER*β*- and ER*α*-positive cells in PHTPP-treated pups compared with those in the HI + ICA + Vehicle group. The western blot results were consistent with the immunofluorescence data (Figures 13(c)–13(e)). In in vitro preliminary experiments, the optimal inhibitory concentration of PHTPP was determined to be 8 *μ*mol/L (Supplementary material 10), and the in vitro results were similar to those above (Supplementary material 11), thus supporting and verifying the findings obtained in vivo. The above experimental results showed that PHTPP treatment effectively inhibited both ER*β* expression in ICA-pretreated HIBD neonatal mice and ER*α*.

**Figure 14:**
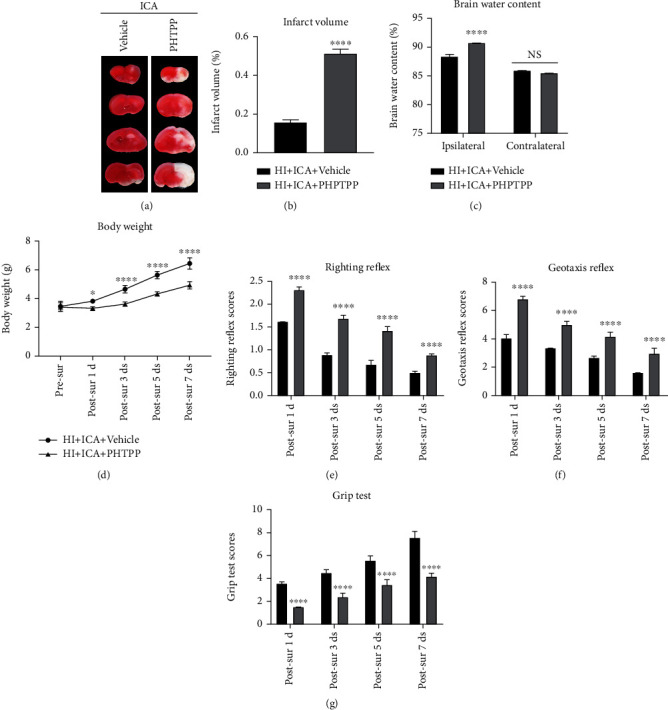
Effects of the ER*β* inhibitor PHTPP on cerebral infarction volume, cerebral water content, and neurological function in HIBD newborn mice. (a) TTC staining photos of brain tissue sections, *n* = 6 mice in each group. (b) Quantitative analysis of cerebral infarct volume shown by TTC staining. (c) Quantitative analysis of brain water content in HIBD pups, *n* = 6 mice in each group. (d) Quantitative analysis of the body weight, (e) righting reflex, (f) negative geotaxis, and (g) grip tests on days 1, 3, 5, and 7 after HIBD induction in neonatal mice, *n* = 8 mice in each group. ^∗^*P* < 0.05 and ^∗∗∗∗^*P* < 0.0001 compared with the HI + ICA + Vehicle group, NS = no significant difference. Data are presented as the mean ± SDs.

We then further explored the neuroprotective role of the ER*β* inhibitor PHTPP in HIBD newborn mice pretreated with ICA. The TTC (Figures 14(a) and 14(b)), brain water content (Figure 14(c)), body weight measurement (Figure 14(d)), and neurobehavioral experiment results (Figures 14(e)–14(g)) were consistent with the data obtained with the autophagy inhibitor 3-MA. We therefore concluded that PHTPP treatment reversed the neuroprotective effect of ICA pretreatment on HIBD newborn mice and exacerbated HIBD. Thus, ER*β* activation might play an important role in the neuroprotection of newborn mice with HIBD.

### 3.9. The ER*β* Inhibitor PHTPP Reversed the Promotion of Autophagy Resulting from ICA Pretreatment in HIBD Newborn Mice

**Figure 15:**
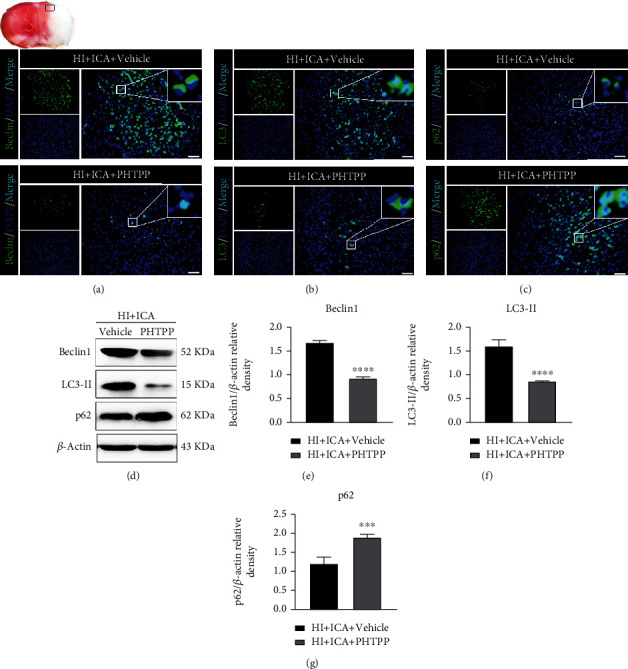
Effects of the ER*β* inhibitor PHTPP on autophagy-related proteins in HIBD neonatal mice pretreated with ICA. The expression of (a) Beclin1, (b) LC3, and (c) p62 in the cerebral ischemic penumbra (black box of the brain slice in the upper left corner) in neonatal HIBD mice treated with PHTPP detected by fluorescence, *n* = 8 mice in each group. Bar = 100 *μ*m. (d) Representative western blot images and (e–g) quantitative analysis. ^∗∗∗^*P* < 0.001, and ^∗∗∗∗^*P* < 0.0001 compared to the HI + ICA + Vehicle group. Data are presented as the mean ± SDs, *n* = 6 mice in each group.

We also tested whether the neuroprotective effect of autophagy activation produced by ICA pretreatment was dependent on ER*β*. Immunofluorescence staining (Figures 15(a)–15(c)) showed that compared with the HI + ICA + Vehicle group, the number of Beclin1- (Figure 15(a)) and LC3-positive cells (Figure 15(b)) in pups treated with PHTPP significantly decreased, and the number of p62-positive cells (Figure 15(c)) significantly increased. The western blot results were consistent with the immunofluorescence data (Figures 13(d)–13(g)). Similar results were achieved with cells in vitro, supporting our findings in vivo (Supplementary material 12). The above results indicated that the ER*β* inhibitor PHTPP significantly reversed the promotion of autophagy brought about by ICA preconditioning in HIBD neonatal mice and inhibited autophagy.

### 3.10. The ER*β* Inhibitor PHTPP Reversed the Inhibition of Apoptosis Resulting from ICA Pretreatment in HIBD Newborn Mice

**Figure 16:**
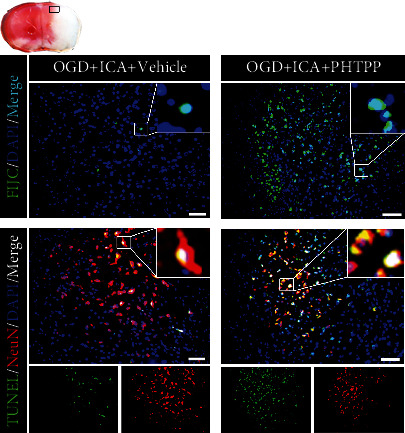
Effects of PHTPP treatment on apoptosis in HIBD neonatal mice pretreated with ICA. The numbers of TUNEL-positive cells (green fluorescence in the lower panel), NeuN-positive cells (red fluorescence), and FJC-positive neurons (green fluorescence in the upper panel) in the cerebral ischemic penumbra (small black box in the upper left brain section) in PHTPP-treated HIBD newborn mice detected by tissue immunofluorescence, *n* = 8 mice in each group. Bar = 100 *μ*m.

**Figure 17:**
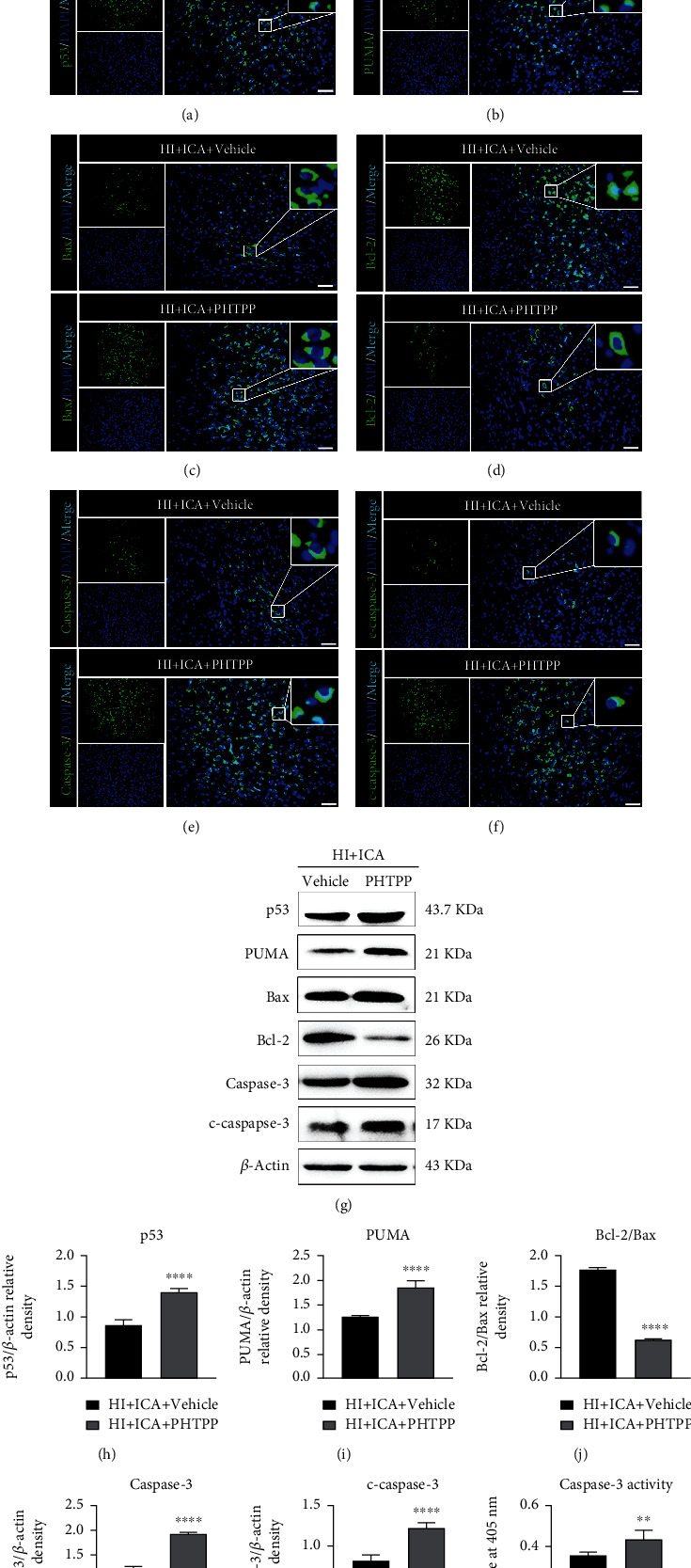
Effects of the ER*β* inhibitor PHTPP on apoptosis-related proteins in HIBD neonatal mice pretreated with ICA. The expression levels of (a) p53, (b) PUMA, (c) Bax, (d) Bcl-2, (e) caspase-3, and (f) cleaved caspase-3 in the cerebral ischemic penumbra (small black box in the upper left brain section) in PHTPP-treated HIBD newborn mice detected by tissue immunofluorescence, *n* = 8 mice in each group. Bar = 100 *μ*m. (g) Representative western blot images and (h–l)quantitative analysis. (m) Quantitative analysis of the detected caspase-3 activity. ^∗∗^*P* < 0.01, and ^∗∗∗∗^*P* < 0.0001 compared to the HI + ICA + Vehicle group. Data are presented as the mean ± SDs, *n* = 6 mice in each group.

Finally, we examined the effect of ER*β* inhibition on the level of apoptosis in HIBD newborn mice pretreated with ICA. TUNEL and FJC staining (Figure 16) showed that compared with the HI + ICA + Vehicle group, the number of TUNEL-positive cells and FJC-positive neurons in PHTPP-treated pups were significantly increased. Tissue immunofluorescence staining (Figures 17(a)–17(f)) showed that compared with that in the HI + ICA + Vehicle group, after PHTPP treatment, the number of p53- (Figure 17(a)), PUMA- (Figures 17(g)–17(l)), Bax- (Figure 17(c)), caspase-3- (Figure 17(e)), and cleaved caspase-3-positive cells (Figure 17(f)) increased significantly. Additionally, the number of Bcl-2-positive cells (Figure 17(d)) was significantly reduced. The western blot results were consistent with the immunofluorescence data (Figures 17(g)–17(l)). Moreover, caspase-3 activity (Figure 17(m)) in pups treated with PHTPP increased significantly. Similar results were achieved with cells in vitro, supporting our findings in vivo (Supplementary material 13). The above data showed that PHTPP treatment significantly promoted apoptosis in HIBD pups and indicated that the ER*β* inhibitor PHTPP reversed the inhibitory effect of ICA pretreatment on apoptosis in HIBD newborn mice and promoted apoptosis.

## 4. Discussion

In this study, we reported the specific mechanism by which ICA exerts a neuroprotective effect through apoptosis inhibition in HIBD newborn mice. Specifically, we have shown that (1) ICA pretreatment significantly promoted autophagy in HIBD newborn mice; (2) the autophagy inhibitor 3-MA significantly inhibited autophagy, reversed the neuroprotective effect of ICA pretreatment on HIBD newborn mice, and aggravated apoptosis in HIBD newborn mice; (3) ICA pretreatment significantly upregulated the expression levels of ER*α* and ER*β* in HIBD newborn mice; (4) the ER*α* inhibitor MPP and ER*β* inhibitor PHTPP had clear inhibitory effects on the increases in the expression levels of the ER*α* and ER*β* proteins activated by ICA pretreatment, which reversed the neuroprotective effect of ICA in HIBD newborn mice, inhibited autophagy in HIBD newborn mice pretreated with ICA, promoted apoptosis, and aggravated HIBD in newborn mice.

The role of autophagy in neonatal HIBD is controversial, and its role in ischemic brain injury appears to depend on animal species, the extent of autophagy induction, and the duration of autophagy activation [31]. In our study, we speculated that the neuroprotective mechanism of ICA on neonatal mouse HIBD might be related to the activation of autophagy. Beclin1, as the initiator of autophagy in mammalian cells, can recruit autophagy-related proteins to be localized on the autophagosome membrane, causing autophagosome membrane nucleation and autophagy precursor membrane prolongation, thus promoting autophagosome maturation and upregulating autophagy [62]. During autophagy, LC3 is a key molecule for vesicle elongation during the formation of autophagosomes. LC3 exists in the cytoplasm and is normally transferred to LC3-I through ATG4-mediated dihydroxylation. When autophagy is activated, LC3-I combines with phosphatidylethanolamine to form LC3-II, which is present in the inner and outer membranes of autophagosomes, marking autophagosome formation [63]. p62 acts as a bridge in the process of autophagy. The LIR domain of p62 can bind to the LC3 protein to form a complex, which is then degraded by autophagosomes and lysosomes [64]. In a rat model of I/R and an HT22 cell OGD/R model, Sun et al. found that eugenol pretreatment significantly promoted the expression of Beclin1, increased the ratio of LC3-II/LC3-I, and reduced the expression level of the p62 protein, indicating that eugenol pretreatment decreased I/R-induced autophagy in rats [65]. Moreover, the authors found that the activation of autophagy helped to reduce the cerebral infarct volume and neurological deficits in I/R rats and that autophagy activation was involved in the neuroprotective effects of eugenol pretreatment in I/R rats. Consistent with the results of Sun et al., the in vitro (Supplementary material 2) and in vivo experimental results in this study showed that when neonatal mice developed HIBD and HT22 cells experienced OGD injury, the expression of Beclin1 and the ratio of LC3-II/LC3-I were significantly decreased, and the expression level of the p62 protein was significantly increased, indicating that autophagy was inhibited during HIBD in neonatal mice. In neonatal mice pretreated with ICA, the expression of Beclin1 and the ratio of LC3-II/LC3-I were significantly increased, and the expression of the p62 protein was significantly decreased. ICA pretreatment significantly promoted autophagy in HIBD neonatal mice. These results suggested that the mechanism by which ICA pretreatment provides neuroprotection to neonatal HIBD might be related to the activation of autophagy and that autophagy signals might play a protective role in the pathogenesis of neonatal HIBD.

Autophagy and apoptosis are closely related cellular physiological processes that have been proven to be jointly involved in the occurrence and development of neonatal HIBD. Research conducted by Yang et al. showed that Dengzhan Xixin injection could improve mitochondrial injury induced by cerebral I/R in SD rats by activating mitochondrial autophagy and inhibiting mitochondrial-mediated apoptosis [66]. Consistent with Yang et al., our study demonstrated that ICA pretreatment could improve HIBD in neonatal mice by activating autophagy and inhibiting the level of apoptosis. However, what are the specific mechanisms of autophagy and apoptosis that are involved in the neuroprotective effects of ICA in neonatal mice with HIBD? Based on previous studies, we speculated that the activation of autophagy might be an upstream event for apoptosis inhibition. The mitochondrial pathway is the main apoptotic factor after cerebral ischemic injury [67]. After the preliminary study on key autophagy proteins, we examined key indicators of mitochondrial pathway-mediated apoptosis for further in-depth study. p53 is a tumor suppressor gene that participates in the regulation of DNA repair, apoptosis, autophagy, proliferation, differentiation, and many other phenotypes. When DNA damage is induced by the stress response, p53 rapidly responds to DNA damage and shifts to the nucleus to trigger the mitochondrial-dependent apoptotic pathway [68]. It has been reported that pifithrin-alpha, an inhibitor of p53, can effectively inhibit PUMA levels and delay neuronal death in the hippocampal CA1 region of SD rats after transient global cerebral ischemic injury, indicating that inhibition of p53 and PUMA expression can effectively improve cerebral ischemic injury [69]. As a key regulator of mitochondrial function, PUMA is a proapoptotic protein in the Bcl-2 family that contains only the BH3 homologous region. PUMA can be highly transcriptionally activated as a transcriptional target of p53 and is mainly responsible for the transmission of mitochondrial death signals [70]. Yu et al. found that the elimination or inhibition of PUMA expression can block apoptotic signaling and improve tissue damage and cell death induced by degenerative diseases [71]. PUMA can induce the activation of the proapoptotic gene Bax through the combination of its BH3 homologous region and the Bcl-2 family antiapoptotic gene Bcl-2. The activation of Bax promotes the formation of pores on the mitochondrial membrane and thus leads to the destruction of the integrity of the mitochondrial outer membrane, which induces the release of mitochondrial cytochrome C into the cytoplasm through the membrane gap and activates the caspase cascade to induce apoptosis [72].

In subsequent studies, we used 3-MA to inhibit autophagy and detected the neuroprotective effects of autophagy inhibition on ICA-pretreated HIBD newborn mice and the impact of mitochondrial pathway-mediated apoptosis levels. It has been reported that activation of autophagy can promote a reduction in the level of apoptosis and alleviate I/R nerve damage in the brains of SD rats [65]. In rats, aspergillin A reduces neuronal apoptosis induced by subarachnoid hemorrhage by activating autophagy and improves neurological deficits [73]. In addition, during I/R in SD rats, ischemic postconditioning improves cerebral I/R injury by activating autophagy, while 3-MA treatment significantly reverses the neuroprotective role of ischemic postconditioning and aggravates apoptosis [74]. Consistent with the mentioned research, in our study, when neonatal HIBD mice were pretreated with ICA and injected with 3-MA via the lateral ventricle, we found that the expression of the autophagy-related protein Beclin1 and the ratio of LC3-II/LC3-I were significantly decreased; the expression of p62 was significantly increased, and the level of autophagy was significantly inhibited. Moreover, treating HIBD newborn mice with 3-MA significantly reversed the neuroprotective effect of ICA pretreatment, increased the cerebral infarct volume and brain water content, significantly reduced the body weight, and aggravated neurological impairment. In addition, we found that the expression levels of the apoptosis-related proteins p53, PUMA, Bax, caspase-3, and cleaved caspase-3 were significantly increased, and the expression level of Bcl-2 was significantly decreased. 3-MA treatment significantly reversed the inhibitory effect of ICA pretreatment on apoptosis in HIBD newborn mice and exacerbated apoptosis. In vitro (Supplementary material 3 and Supplementary material 4), 3-MA treatment had the same damaging effect on OGD-injured HT22 cells pretreated with ICA. Therefore, we believe that the neuroprotective effect of ICA pretreatment on apoptosis inhibition in neonatal mice with HIBD may be mediated by autophagy activation.

Finally, we investigated the specific molecular mechanism by which ICA pretreatment activates autophagy and inhibits apoptosis after HIBD in neonatal mice. It has been reported that endogenous estrogen maintains the expression of antiapoptotic-related proteins through the ER*α* signaling pathway to alleviate I/R injury in the brains of SD rats [75]. Guanxin Danshen formulation alleviated myocardial I/R injury in SD rats by upregulating ER*β* expression levels [76]. Selective activation of ER*β* helps to improve the neuroinflammatory injury induced by brain I/R in postmenopausal women [77]. In addition, phytoestrogen isoflavones can reduce ischemic stroke injury by interfering with glutamate oxaloacetate transaminase [78]. Consistent with the mentioned research, in our study, when neonatal mice developed HIBD, the expression levels of ER*α* and ER*β* in the brains of neonatal mice were significantly decreased, and pretreatment with ICA, a medicinal plant with estrogen-like effects, significantly increased the levels of ER*α* and ER*β* in the brains of neonatal mice with HIBD. We speculated that the upregulation of ER*α* and ER*β* mediated the molecular mechanism by which ICA exerts its neuroprotective effects in HIBD newborn mice. However, there is controversy surrounding previous reports as to whether ER*α* and ER*β* have neuroprotective effects in brain injury. Some researchers believe that the ER exerts a neuroprotective effect on cerebral ischemic injury mainly through ER*α*. For example, Dubal et al. found that the neuroprotective effect of E2 on cerebral I/R injury mice was eliminated in ER*α*-knockout mice, while ER*β*-knockout mice maintained the E2-mediated neuroprotective effect [79]. In addition, the ER*α* agonist propyl pyrazole triol can protect hippocampal CA1 neurons from synaptic dysfunction caused by cerebral ischemic injury, while the ER*β* agonist diaryl propionitrile has not yet been effective [80]. The above studies show that E2 exerts a neuroprotective effect on animal cerebral I/R injury mainly through ER*α* rather than ER*β*. However, additional research has found that the neuroprotective effect of the ER on animals with ischemic brain injury is mediated by ER*β* rather than ER*α*. For example, Carswell et al. showed that diaryl propionitrile, an ER*β* agonist, exhibited a significant neuroprotective effect in a global cerebral ischemia model, while propyl pyrazole triol, an ER*α* agonist, did not affect neuronal injury induced by global cerebral ischemia [81]. Long-term regular E2 treatment can protect SD rats from cognitive dysfunction and learning and memory deficits induced by cerebral ischemic injury, while ER*β* silencing reverses E2-mediated neuroprotection and aggravates cerebral ischemic injury [82]. Although these studies are contradictory, they show the importance of ER*α* and ER*β* in the survival of ischemic neurons from different aspects. However, some studies have negated the neuroprotective effects of estrogen. In an aged mouse model of cerebral I/R, studies have shown that exogenous estrogen treatment does not produce neuroprotection after brain injury in aged postmenopausal female mice [83]. Based on the above findings, we believe that the neuroprotective effects of ER*α* and ER*β* on cerebral ischemia injury may be affected by multiple factors. Among them, the degree of brain injury, animal species, animal model, route and mode of treatment different estrogen levels, and sampling time may affect the ER*α*- and ER*β*-mediated neuroprotection [84]. In our study, we found that the expression level of ER*β* was also significantly decreased after the ER*α* inhibitor MPP reduced the expression level of ER*α* in ICA-pretreated HIBD neonatal mice (in vitro results are shown in Supplementary material 7), as did PHTPP (in vitro results are shown in Supplementary material 1). In our study, we demonstrated that the ER*α* inhibitor MPP can reverse the neuroprotective effect of ICA preconditioning in HIBD neonatal mice, thereby exacerbating HIBD. We also demonstrated that the expression level of ER*β* was significantly decreased when neonatal mice developed HIBD but significantly increased after ICA administration. Therefore, we believe that decreased ER*β* expression caused by the ER*α* inhibitor MPP may be indirectly induced by aggravated HIBD injury in neonatal mice. In addition, it has been reported that ER*α* and ER*β* are similar in their structure and ability to bind to estradiol and coexist in a small number of cells. ER*α* and ER*β* may also interact and influence each other, and both participate in the occurrence and development of HIBD.

In the present study, we confirmed that ICA pretreatment can play a neuroprotective role in neonatal mouse HIBD by promoting autophagy and inhibiting apoptosis. Additionally, we found that ICA pretreatment upregulated the expression of ER*α* and ER*β* in HIBD neonatal mice. However, does the molecular mechanism by which ICA pretreatment offers neuroprotection by promoting autophagy and inhibiting apoptosis in neonatal mice with HIBD depend on ER*α* and ER*β* mediation? We carried out further research on this topic. Previous studies have shown that ER*α* and ER*β* have significant regulatory effects on autophagy and apoptosis. Neuroprotective miR-7-1 plays a functional neuroprotective role by enhancing EST (an ER*α* and ER*β* agonist) to inhibit neuronal apoptosis induced by spinal cord injury [85]. The overexpression of ER*α* and ER*β* provides neuroprotection by upregulating antiapoptotic-related proteins to inhibit apoptosis induced by spinal cord injury [86]. In addition, in an atherosclerotic mouse model, the phytoestrogen dioscin showed a protective role by promoting autophagy and inhibiting postmenopausal atherosclerosis-induced apoptosis through the peroxisome proliferator-activated receptor *γ* coactivator alpha (PGC-1*α*)/ER*α* pathway [87]. Consistent with the above studies, herein, we found that when ICA was administered to HIBD newborn mice at the same time as intracerebroventricular injection of the ER*α* inhibitor MPP and ER*β* inhibitor PHTPP, the neuroprotective effects of ICA pretreatment were significantly reversed; the cerebral infarct volume and brain water content were increased; the body weights were significantly reduced, and neurological impairment was significantly aggravated. Moreover, we found that the expression level of the autophagy-related protein Beclin1 and the ratio of LC3-II/LC3-I were significantly decreased, and the expression level of p62 was significantly increased. Additionally, the expression levels of the apoptosis-related proteins p53, PUMA, Bax, caspase-3, and cleaved caspase-3 were significantly increased, while the expression level of Bcl-2 was significantly decreased. Inhibition of ER*α* and ER*β* significantly reversed the neuroprotective effect of ICA pretreatment in HIBD newborn mice, resulting in autophagy inhibition and apoptosis promotion. In vitro (Supplementary material 8, Supplementary material 9, Supplementary material 12 and Supplementary material 13), inhibiting ER*α* and ER*β* caused the same damage to OGD-injured HT22 cells pretreated with ICA. Therefore, we believe that the molecular mechanism by which ICA pretreatment offers neuroprotection to mice with HIBD by promoting autophagy and then inhibiting apoptosis may be mediated by the activation of ER*α* and ER*β* pathways. Our findings are helpful to enrich and deepen our understanding of the neuroprotective mechanism of ICA.

The purpose of this study was to investigate whether ICA preconditioning promotes autophagy by activating the ER*α* and ER*β* pathways and then inhibits HIBD-induced apoptosis to exert neuroprotective effects in neonatal mice with HIBD. Other key transcription factors that mediate neuroprotection, such as nuclear factor kappa B (NF-*κ*B), inhibition of kappa B kinase-alpha (IKK-*α*), and granulocyte colony-stimulating factor (G-CSF/CSF3) may also be key pathways by which ICA exerts neuroprotective effects. The transcription factors in the NF-*κ*B family are the main transcription factors involved in inflammatory gene expression; moreover, these transcription factors regulate a variety of cytokines during central nervous system injury and play an important role in its treatment. IKK-*α* is the main subunit of the transcription factor activator protein IKK, and the phosphorylation of IKK-*α* is important for the activation of NF-*κ*B. Focusing on the IKK/NF-*κ*B pathway as a way to regulate the neuroinflammatory injury induced by hypoxia-ischemia may be a promising therapeutic strategy to reduce HIBD in neonatal mice [88]. Studies have shown that ICA can exert a neuroprotective effect on neuroinflammation induced by microglial activation by inhibiting the IKK/NF-*κ*B pathway [89]. Narciclasine attenuates the secretion of proinflammatory factors by downregulating the Akt/IKK/NF-*κ*B signaling pathway and directly inhibits the catalytic activity of IKK-*α* to offer neuroprotection against lipopolysaccharide-induced neuroinflammation [90]. In a rat cerebral I/R model, ICA enhanced mild hypothermia-induced neuroprotection by inhibiting NF-*κ*B activation [91]. However, in the neonatal mouse HIBD model, whether ICA can modulate neuroinflammation induced by neonatal mouse HIBD via the IKK/NF-*κ*B pathway is unknown. In addition, whether ICA promotes autophagy and inhibits apoptosis may depend on mediation of the IKK/NF-*κ*B signaling pathway and whether there is an upstream-downstream or parallel relationship between IKK/NF-*κ*B and ER*α* and ER*β* are worth investigating in the future. Furthermore, we found that the potent neuroprotective agent CSF3 attenuates neuroinflammation and neuronal apoptosis by modulating inflammatory and apoptotic mediators and promotes neurogenesis and angiogenesis, thereby playing a neuroprotective role in childhood brain injury [92]. In an experimental model of optic nerve ischemia, ICA induced endogenous CSF3 production in retinal cells by binding to the transcription factor CEBP-*β* [93]. CSF3 phosphorylation of Akt inactivates the canonical NF-*κ*B pathway and inhibits the production of proinflammatory cytokines (PICs) and nitric oxide (NO), further reducing neuroinflammation. Thus, CSF3 may be a key upstream molecule by which ICA regulates the IKK/NF-*κ*B pathway to inhibit neuroinflammation [94]. In the next study, we will focus on G-CSF/IKK/NF-*κ*B-mediated neuroinflammation and apoptosis and the neuroprotective molecular mechanism of the pharmacological effects of ICA. Additionally, the neuroprotective mechanisms mediated by two important pathways that are the pharmacodynamic basis of ICA, the phosphoinositide 3-kinase/protein kinase B (PI3K-Akt) and nuclear red blood cell 2-related factor 2 (Nrf-2) pathways, may also have neuroprotective effects on neonatal mouse HIBD, which is worthy of exploration in future research [95]. A preprint has previously been published [96].

## 5. Conclusion

ICA pretreatment may promote autophagy by activating the ER*α* and ER*β* pathways and then inhibit HIBD-induced apoptosis to play a neuroprotective role in neonatal HIBD mice. Our study has provided new insights into the neuroprotective mechanisms of ICA pretreatment on HIBD in neonatal mice.

## Data Availability

All data and materials are available on request from authors.

## Supplementary Materials

Supplementary Materials To make the article concise and clear, we consider putting the results of in vitro experiments into supplementary materials to support the conclusions of in vivo experiments, and the data of our in vivo experiments are sufficient to support our conclusions in each part. Please refer to the supplementary materials for results and description of all in vitro experiments.
