# Supplementary material for: Neuroprotective Mechanism of Icariin on Hypoxic Ischemic Brain Damage in Neonatal Mice
Source: Oxid Med Cell Longev. 2022 Nov 15;2022:1330928. doi: 10.1155/2022/1330928 (PMC9681555; doi:10.1155/2022/1330928)
Supplement: Supplementary Materials — To make the article concise and clear, we consider putting the results of in vitro experiments into supplementary materials to support the conclusions of in vivo experiments, and the data of our in vivo experiments are sufficient to support our conclusions in each part. Please refer to the supplementary materials for results and description of all in vitro experiments. [file 1330928.f1.zip › Supplementary material 1 (1).docx]

**Supplementary material 1**

To determine the optimal hypoxia time of OGD and the optimal dose of ICA, CCK8 assays were used to determine the survival rate of HT22 cells in this study. The results of the CCK8 experiment (Figure A) showed that, compared with the control group, cell viability decreased in a time-dependent manner when OGD was applied for 4, 6, and 8 h. After 4 h of OGD, cell viability was approximately 60%. Therefore, 4 h of OGD was selected for the concentration experiment. In an attempt to determine the optimal dose of ICA to be administered (Figure B), HT22 cell activity was determined and found to be approximately 90% after treatment with 8 μmol/L ICA when compared to the OGD group. Therefore, 4 h of OGD and 8 μmol/L ICA were selected as the optimal hypoxia time of OGD and the optimal administered dose of ICA, respectively. Moreover, the toxicity of ICA to HT22 cells was determined. The experimental results (Figure C) showed that different doses/concentrations of ICA caused no toxicity to HT22 cells, reflecting the safety of ICA administration to a certain extent. To verify the success of the model and the effect of 8 μmol/L ICA, the HT22 cytoskeletal protein βIII-tubulin was labeled by immunofluorescence. The results (Figure D) showed that the cells in the control group were in good shape, and the cell bodies were large, triangular, or polygonal. After OGD injury, the cell bodies decreased in size, and most cells showed a round or spindle-shaped shrunken morphology, proving that the model was established successfully. Compared with the OGD + Vehicle group, ICA pretreatment significantly improved HT22 cell injury and significantly restored their morphology, indicating that 8 μmol/L ICA pretreatment had significant neuroprotective effects on OGD-injured HT22 cells.

**
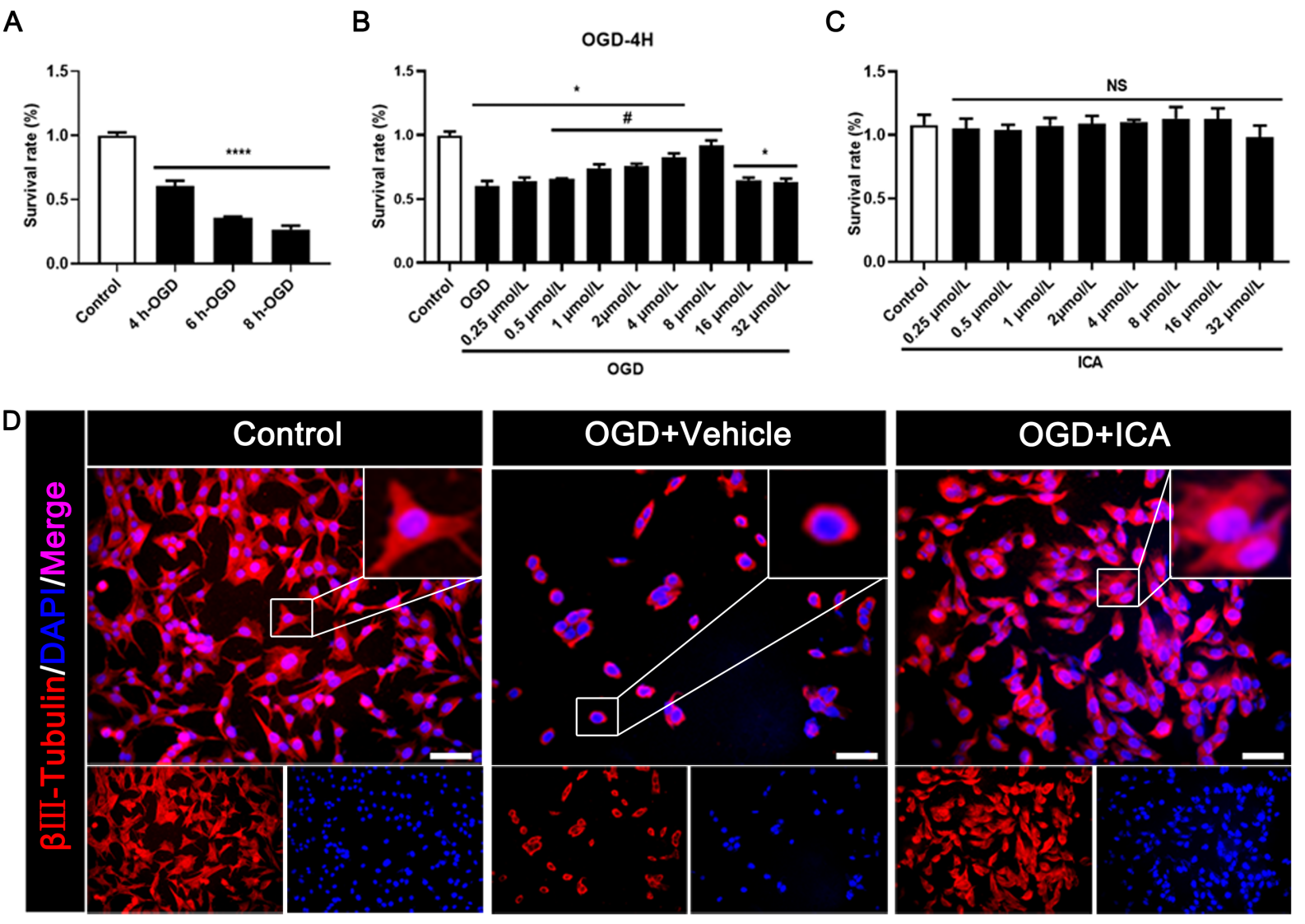
**

**Figure:** Detection of OGD time, ICA drug concentration and drug toxicity in HT22 cells. (A) The survival rate of HT22 cells was observed at 4 h, 6 h and 8 h after OGD injury, n = 6 in each group. (B) HT22 cell viability after 4 h of OGD injury after ICA pretreatment at concentrations of 0, 0.25, 0.5, 1, 2, 4, 8, 16, and 32 μmol/L. (C) Survival rates of normal HT22 cells after treatment with different concentrations of ICA. (D) Skeletal protein βIII-tubulin fluorescence staining. ^*^*P* < 0.05 and ^****^*P* < 0.0001 vs. the control group, ^#^*P* < 0.05 vs. the OGD + Vehicle group, NS = no significant difference. Bar = 100 μm.
