# Supplementary material for: Neuroprotective Mechanism of Icariin on Hypoxic Ischemic Brain Damage in Neonatal Mice
Source: Oxid Med Cell Longev. 2022 Nov 15;2022:1330928. doi: 10.1155/2022/1330928 (PMC9681555; doi:10.1155/2022/1330928)
Supplement: Supplementary Materials — To make the article concise and clear, we consider putting the results of in vitro experiments into supplementary materials to support the conclusions of in vivo experiments, and the data of our in vivo experiments are sufficient to support our conclusions in each part. Please refer to the supplementary materials for results and description of all in vitro experiments. [file 1330928.f1.zip › Supplementary material 10 (1).docx]

**Supplementary material 10**

To determine the optimal inhibitory concentration of PHTPP to be administered to ICA-pretreated OGD-injured HT22 cells, western blotting was used to detect ERβ protein expression after treatment with different doses of PHTPP. The western blot experimental results (Figure A-B) showed that compared with the control group, all of the different doses of PHTPP showed significant inhibition of ERβ protein expression levels in HT22 cells. Notably, the inhibition effect was most significant at 8 μmol/L. Therefore, we selected 8 μmol/L PHTPP as the best inhibitory concentration. In addition, we determined the toxicity of different doses of PHTPP to HT22 cells by CCK8 assays. The results (Figure C) showed that compared with the control group, the different doses of PHTPP had no significant effect on the survival rate of HT22 cells, and therefore, no cytotoxicity. Thus, 8 μmol/L PHTPP was administered to ICA-pretreated OGD-damaged HT22 cells.


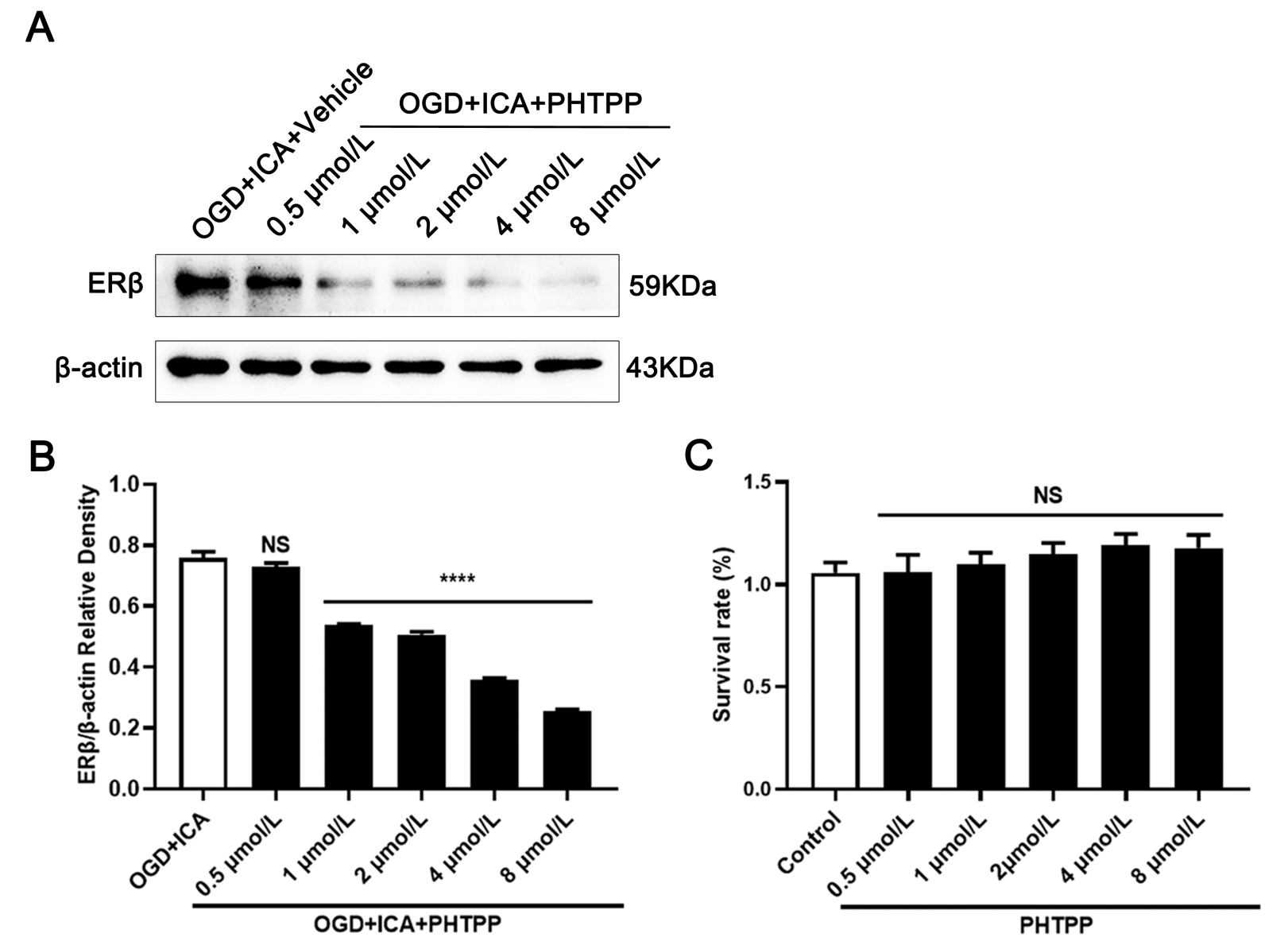


**Figure:** Optimal concentration of the inhibitor PHTPP and cytotoxicity determination. Representative western blot images (A) and quantitative analysis (B) of ERβ in OGD-injured HT22 cells pretreated with ICA administered different doses of PHTPP. (C) Quantitative analysis of the survival rates of normal HT22 treated with different doses of PHTPP. ^****^*P* < 0.0001 compared with the OGD + ICA + Vehicle group, NS = no significant difference. Data are presented as the mean ± SDs.
