# Supplementary material for: Neuroprotective Mechanism of Icariin on Hypoxic Ischemic Brain Damage in Neonatal Mice
Source: Oxid Med Cell Longev. 2022 Nov 15;2022:1330928. doi: 10.1155/2022/1330928 (PMC9681555; doi:10.1155/2022/1330928)
Supplement: Supplementary Materials — To make the article concise and clear, we consider putting the results of in vitro experiments into supplementary materials to support the conclusions of in vivo experiments, and the data of our in vivo experiments are sufficient to support our conclusions in each part. Please refer to the supplementary materials for results and description of all in vitro experiments. [file 1330928.f1.zip › Supplementary material 11 (1).docx]

**Supplementary material 11**


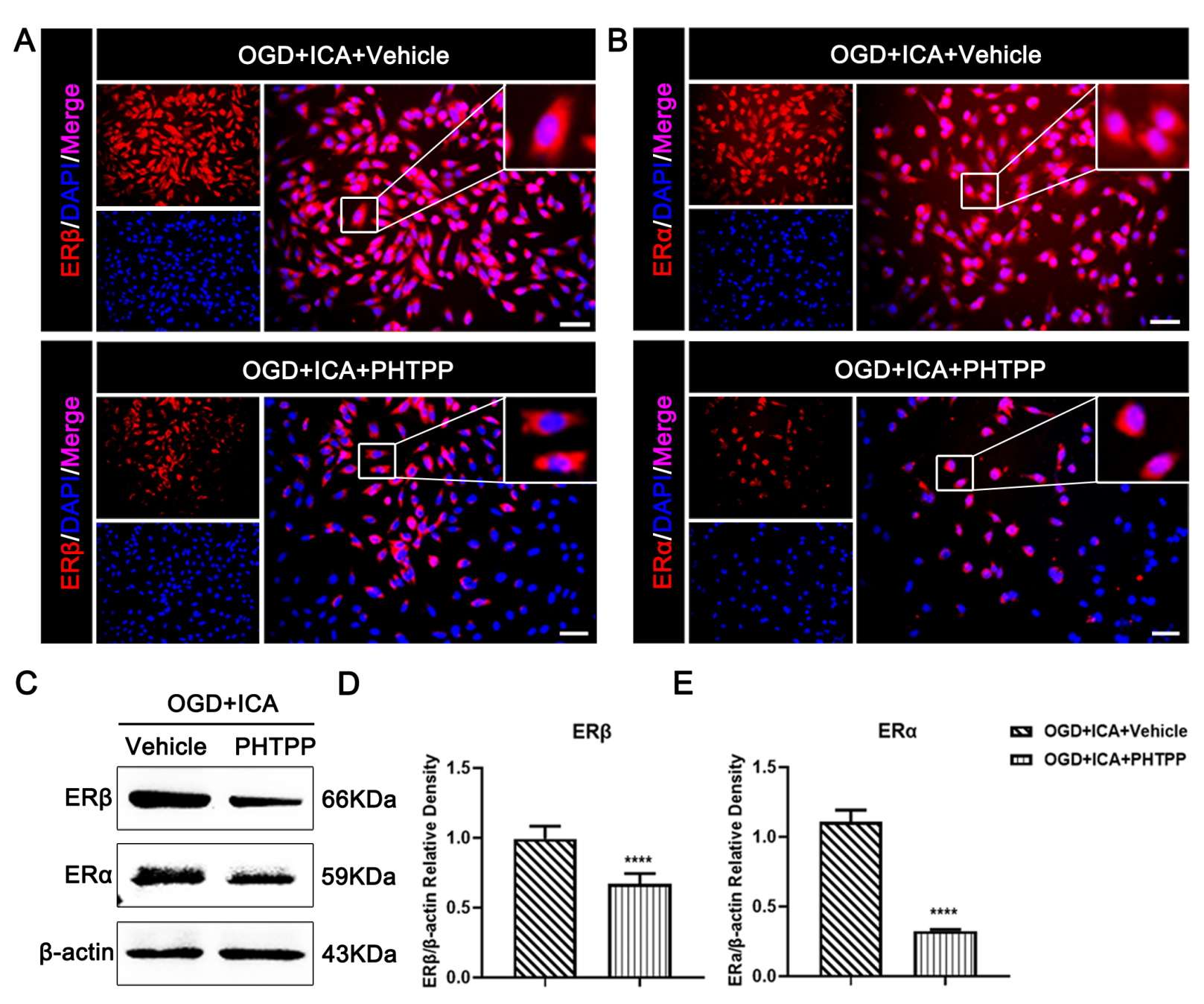


**Figure:** Effect of the ERβ inhibitor PHTPP on ERβ and ERα expression in OGD-injured HT22 cells treated with ICA. The expression levels of ERβ (A) and ERα (B) in HT22 cells damaged by OGD after PHTPP treatment detected by immunofluorescence. Bar = 100 μm. Representative western blot images (A) and quantitative analysis (D-E). ^****^*P* < 0.0001 compared to the HI + ICA + Vehicle group. Data are presented as the mean ± SDs.
