# Supplementary material for: Neuroprotective Mechanism of Icariin on Hypoxic Ischemic Brain Damage in Neonatal Mice
Source: Oxid Med Cell Longev. 2022 Nov 15;2022:1330928. doi: 10.1155/2022/1330928 (PMC9681555; doi:10.1155/2022/1330928)
Supplement: Supplementary Materials — To make the article concise and clear, we consider putting the results of in vitro experiments into supplementary materials to support the conclusions of in vivo experiments, and the data of our in vivo experiments are sufficient to support our conclusions in each part. Please refer to the supplementary materials for results and description of all in vitro experiments. [file 1330928.f1.zip › Supplementary material 2 (1).docx]

**Supplementary material 2**


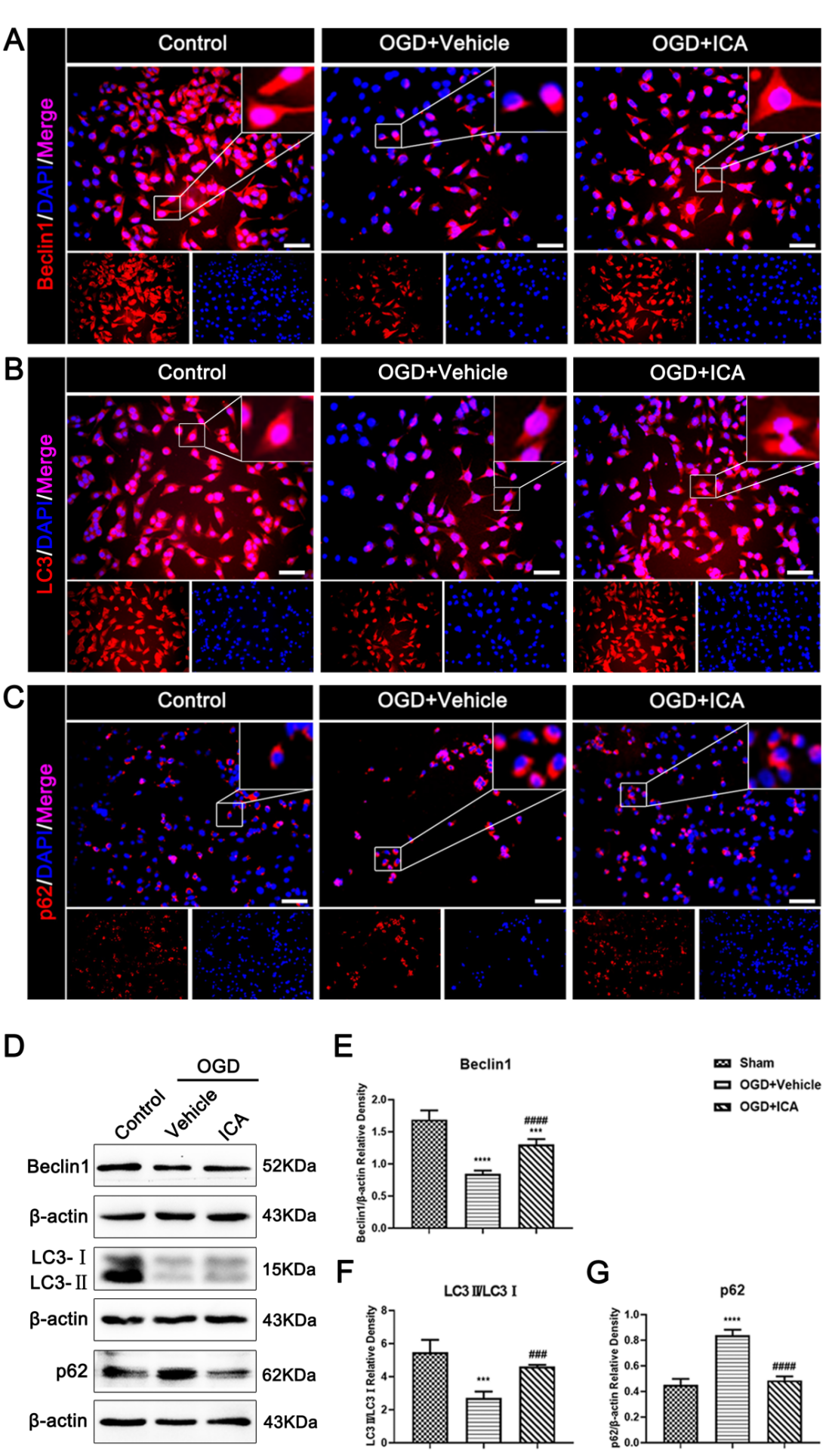


**Figure:** Effect of ICA pretreatment on autophagy in OGD-injured HT22 cells. The expression levels of Beclin1 (A), LC3 (B), and p62 (C) in HT22 cells damaged by OGD after ICA pretreatment detected by immunofluorescence. Representative western blot images (D) and quantitative analysis (E-G). ^***^*P* < 0.001 and ^****^*P* < 0.0001 compared to the control group, ^###^*P* < 0.001 and ^####^*P* < 0.0001 compared to the OGD + Vehicle group. Data are expressed as the means ± SDs.
