# Supplementary material for: Neuroprotective Mechanism of Icariin on Hypoxic Ischemic Brain Damage in Neonatal Mice
Source: Oxid Med Cell Longev. 2022 Nov 15;2022:1330928. doi: 10.1155/2022/1330928 (PMC9681555; doi:10.1155/2022/1330928)
Supplement: Supplementary Materials — To make the article concise and clear, we consider putting the results of in vitro experiments into supplementary materials to support the conclusions of in vivo experiments, and the data of our in vivo experiments are sufficient to support our conclusions in each part. Please refer to the supplementary materials for results and description of all in vitro experiments. [file 1330928.f1.zip › Supplementary material 5 (1).docx]

**Supplementary material 5**


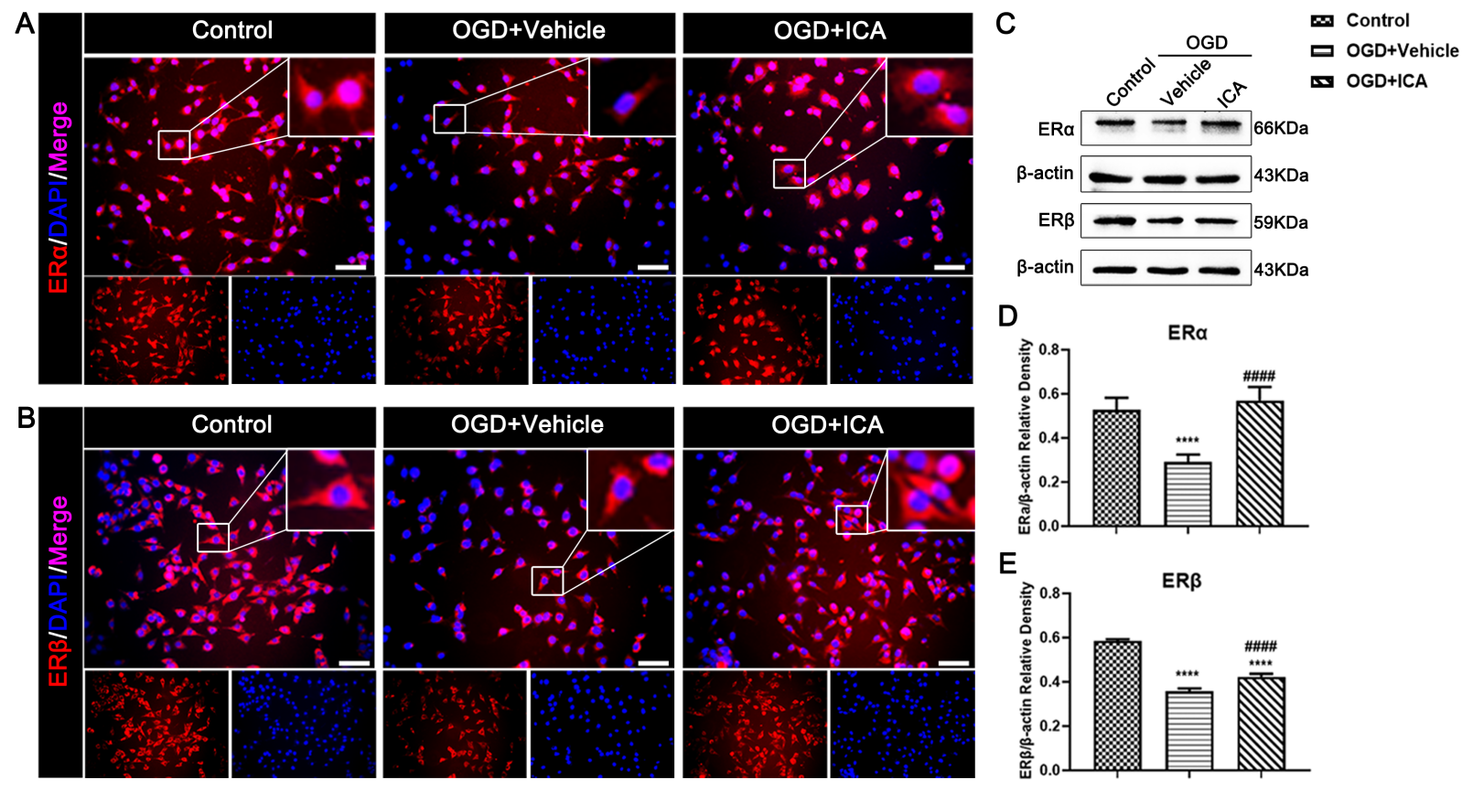


**Figure:** Effects of ICA pretreatment on ERα and ERβ levels in HT22 cells injured by OGD. Immunofluorescence was used to detect the expression of ERα (A) and ERβ (B) in HT22 cells damaged by OGD after ICA pretreatment. Bar = 100 μm. Representative western blot images (C) and quantitative analysis (D and E). ^***^*P* < 0.001 vs. Control group, ^####^*P* < 0.0001 vs. OGD + Vehicle group. Data are presented as the mean ± SDs.
