# Supplementary material for: Neuroprotective Mechanism of Icariin on Hypoxic Ischemic Brain Damage in Neonatal Mice
Source: Oxid Med Cell Longev. 2022 Nov 15;2022:1330928. doi: 10.1155/2022/1330928 (PMC9681555; doi:10.1155/2022/1330928)
Supplement: Supplementary Materials — To make the article concise and clear, we consider putting the results of in vitro experiments into supplementary materials to support the conclusions of in vivo experiments, and the data of our in vivo experiments are sufficient to support our conclusions in each part. Please refer to the supplementary materials for results and description of all in vitro experiments. [file 1330928.f1.zip › Supplementary material 6 (1).docx]

**Supplementary material 6**

To determine the optimal inhibitory concentration of the ERα inhibitor MPP to be administered after OGD-injured HT22 cells were pretreated with ICA, western blotting was used to detect the expression level of the ERα protein after treatment with different doses of MPP. The western blot experiment results (Figure A-B) showed that compared with the control group, all of the different doses of MPP showed significant inhibition of ERα protein expression in HT22 cells damaged by OGD and pretreated with ICA, and the effect was most significant at 1 μmol/L. Therefore, we selected 1 μmol/L MPP as the optimal inhibitory concentration. In addition, CCK8 assays were used to determine the toxicity of different doses of MPP to HT22 cells. The experimental results showed that compared with the Control group, all doses of MPP had no significant effect on the survival rate of HT22 cells (Figure C), and therefore, no cytotoxicity. Thus, we used 1 μmol/L MPP to treat OGD-injured HT22 cells pretreated with ICA.


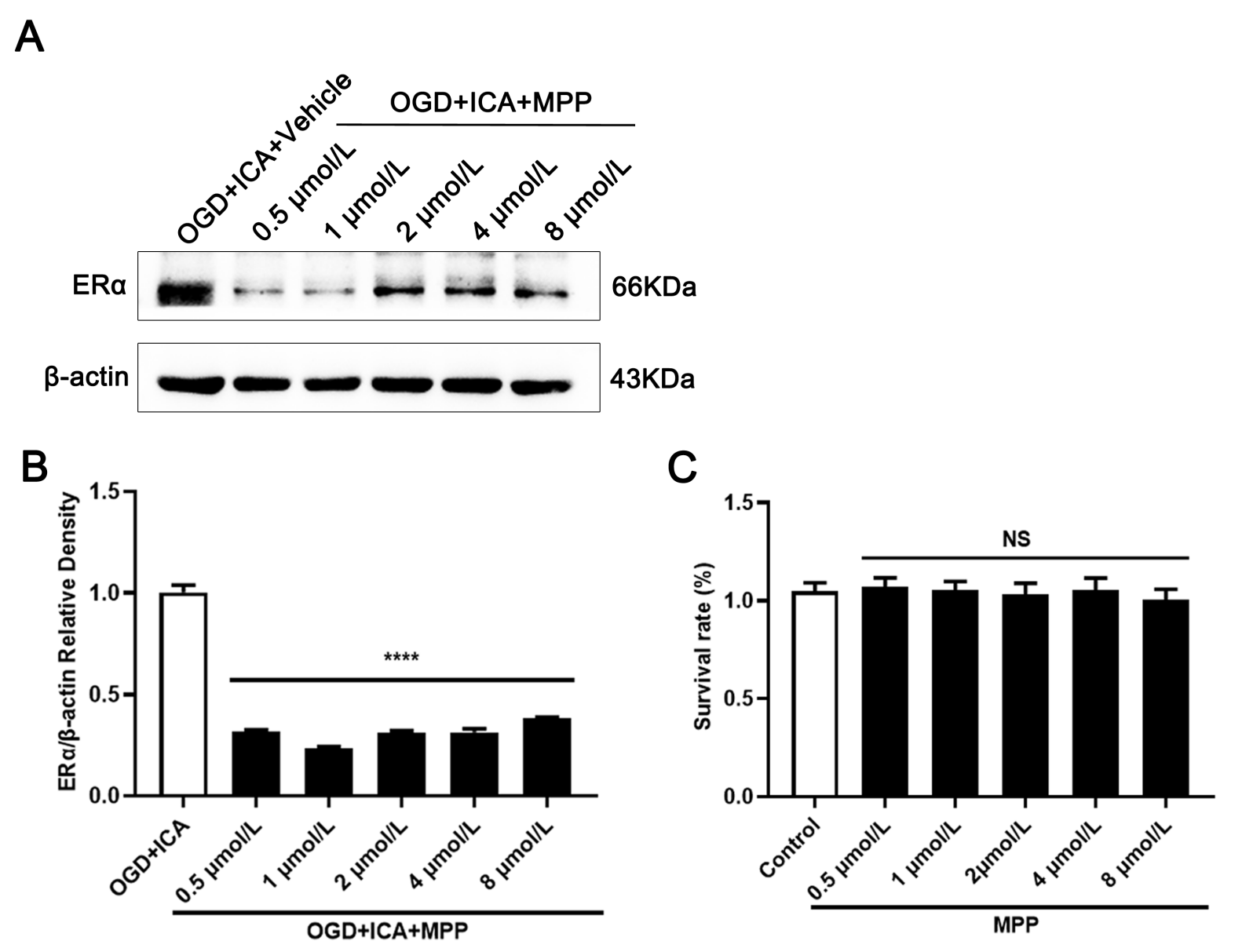


**Figure:** Optimal concentration of the inhibitor MPP and cytotoxicity determination. Representative western blot images (A) and quantitative analysis (B) of ERα in OGD-injured HT22 cells pretreated with ICA and treated with different doses of MPP. (C) Quantitative analysis of the survival rates of normal HT22 cells after treatment with different doses of MPP. ^****^*P* < 0.0001 compared to the OGD + ICA + Vehicle group, NS = not significantly different. Data are presented as the mean ± SDs.
