# Supplementary material for: Neuroprotective Mechanism of Icariin on Hypoxic Ischemic Brain Damage in Neonatal Mice
Source: Oxid Med Cell Longev. 2022 Nov 15;2022:1330928. doi: 10.1155/2022/1330928 (PMC9681555; doi:10.1155/2022/1330928)
Supplement: Supplementary Materials — To make the article concise and clear, we consider putting the results of in vitro experiments into supplementary materials to support the conclusions of in vivo experiments, and the data of our in vivo experiments are sufficient to support our conclusions in each part. Please refer to the supplementary materials for results and description of all in vitro experiments. [file 1330928.f1.zip › Supplementary material 9 (1).docx]

**Supplementary material 9**


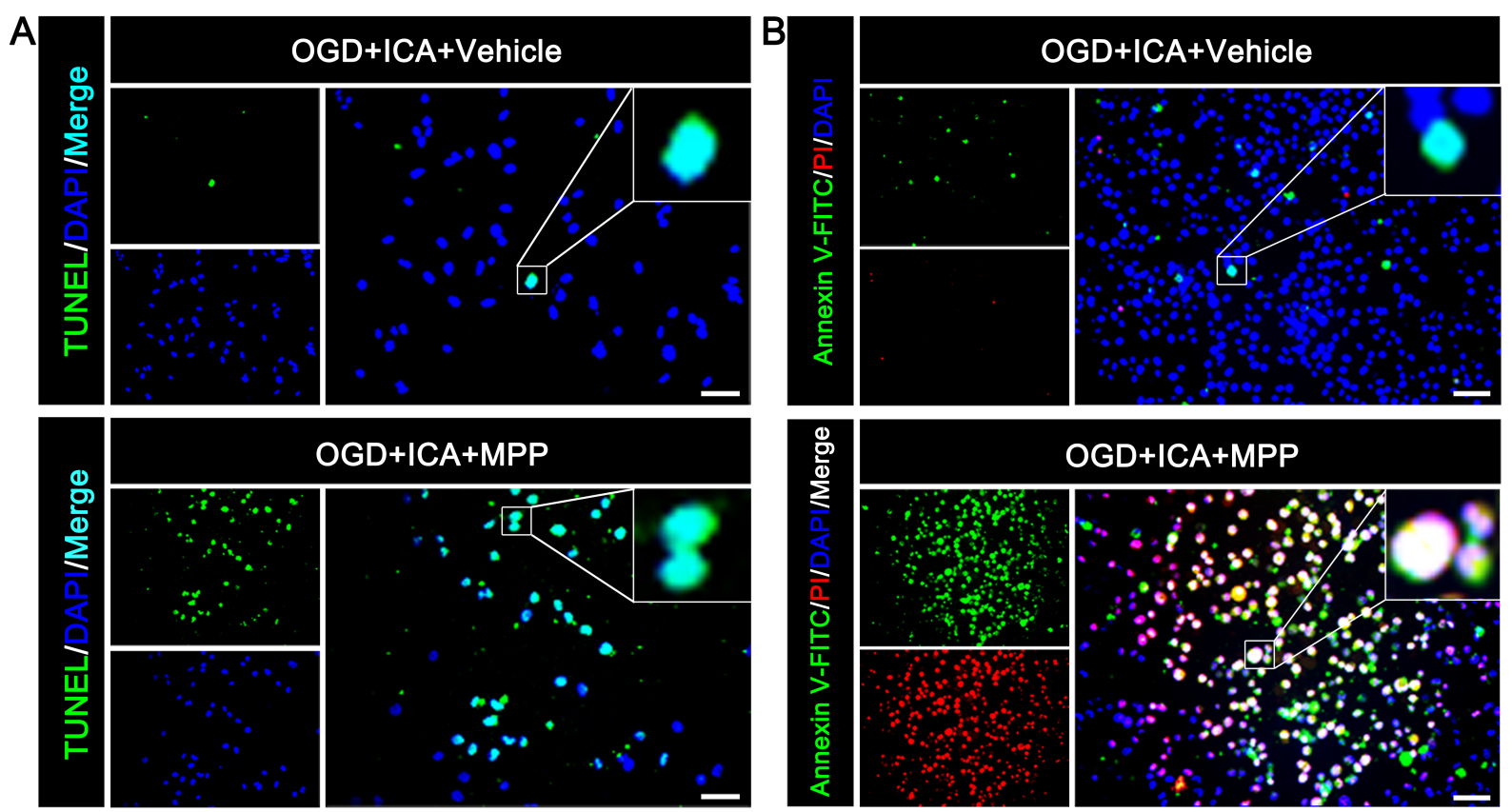


**Figure:** Effect of the ERα inhibitor MPP on the apoptosis of HT22 cells injured by OGD after preconditioning with ICA. The numbers of TUNEL-positive cells (A), Annexin V-FITC-positive cells (B, green fluorescence denotes early apoptotic cells) and PI-positive cells (B, red fluorescence denotes late apoptotic cells and necrotic cells) damaged by OGD after MPP treatment detected by cellular immunofluorescence. Bar = 100 μm.


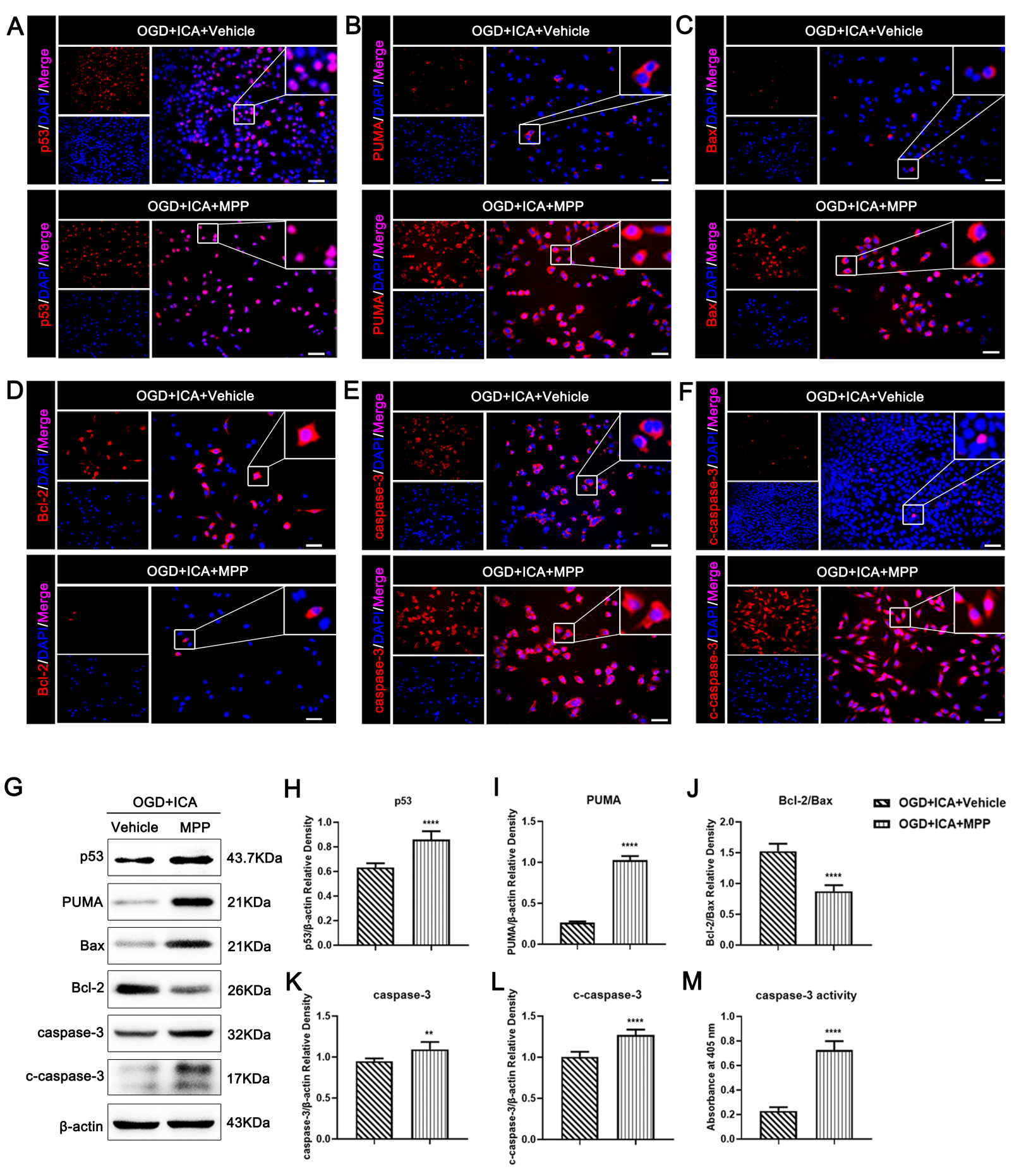


**Figure :** Effect of ERα inhibitor MPP on ICA pretreatment of OGD-injured HT22 cells with apoptosis-associated proteins. The expression levels of p53 (A), PUMA (B), Bax (C), Bcl-2 (D), caspase-3 (E), and cleaved-caspase-3 (F) in HT22 cells damaged by OGD under the experimental conditions of MPP treatment were detected by cellular immunofluorescence. Bar = 100 μm. Representative western blot images (G) and quantitative analysis (H-L). Quantitative analysis results of caspase-3 activity detection (M). ^**^*P* < 0.01, and ^****^*P* < 0.0001 compared to OGD + ICA + Vehicle group. Data are presented as mean ± SDs.
